# Supplementary material for: Making conferences in the plant sciences more inclusive through community recommendations
Source: eLife. 2025 Aug 20;14:e106877. doi: 10.7554/eLife.106877 (PMC12367295; doi:10.7554/eLife.106877)
Supplement: Supplementary file 1. [file elife-106877-supp1.pdf]

December 2022

Inclusive Conferences Working Group Recommendations

The Inclusive Conferences Working Group (ICWG) (part of the ROOT & SHOOT RCN project) was formed to generate an RCN-wide system that develops and implements practices and policies that promote a safe and inclusive conference environment. The ICWG has a charge to generate recommendations, codes, and guidelines relevant to this goal for all seven societies.

Over the last several months the ICWG, which includes over 20 members representing all seven RCN plant science societies, developed a document that details important recommendations that support inclusive and accessible conference environments. The document includes five sections covering recommendations for:

1) An RCN-wide **Community Agreement** for conferences and participants, outlining principles and standards that support equity and inclusiveness,
2) A **reporting structure** that ensures accountability and compliance with the Community Agreement,
3) A **transparent site selection** process that incorporates considerations regarding safety and inclusion of all potential attendees,
4) Guidelines for practices that improve **conference accessibility**, including for those with disabilities and young families,
5) Guidelines for **inclusive selection of speakers and equitable programming**.

We are reaching out to you as volunteers from your organization willing to provide your review of these documents for completeness and provide feedback on areas that could be improved, with an eye toward implementation at your society’s conference. Note that ROOT & SHOOT views these as ‘living documents’, that can be revisited on a regular basis as our communities move toward a future of equity and inclusion.

We would like to finalize this work such that 2023 conferences sponsored by our participating societies can implement ICWG recommendations as appropriate. We request that you review the document in its entirety; the review can be divided into subsections among your volunteer group. Once the ICWG receives feedback from each society's volunteer review team, we will work to generate a final, integrated document and develop a functional recommendation checklist. We acknowledge your time and service as a volunteer reviewer, and your feedback is greatly appreciated

Table of Contents

Community Agreement 2

I. Goal of the community agreement 2

II. Community Values 2

III. Conference Participant Agreement 2

Appendices and links to reporting documents 3

Reporting Structure 4

Key Points 4

1. Pre-Conference Reporting and Feedback 5

2. Reporting and Feedback During a Conference 6

3. Post-Conference Reporting and Feedback 9

Appendix 11

Transparent Site Selection 13

Overview 13

Overarching 13

Sustenance 13

Location setting 14

Venue 14

Transportation 15

Conference Accessibility 16

INTRODUCTION 16

(1) ECONOMIC ENVIRONMENT 17

(2) PHYSICAL ENVIRONMENT 23

(3) AUDIO/VISUAL ENVIRONMENT 25

(4) FAMILY ENVIRONMENT 26

(5) WELLNESS ENVIRONMENT 28

Appendix A: Presentation Guidelines 30

Appendix B: Lactation room characteristics 31

Appendix C: Economic Accessibility 32

Appendix D: Wellness Accessibility - Catering 33

Speaker Selection & Equitable Programming 34

Goals of the document 34

1. Make a Commitment 34

2. Form a Diverse Organizing Committee 34

3. Assess equity goals 35
4. Take care when choosing topics 35
5. Create an equitable schedule 35
6. Provide necessary accessibility options to speakers 36
7. Identify and invite a diverse set of speakers for plenary or keynote sessions 36
8. Develop an equitable criteria for selected talks 36
9. Factor in time for planning 37
10. Be intentional in speaker outreach 37
11. Be respectful of modes of address 37
List of Resources 38

Community Agreement

Inclusive Conferences Working Group A

I. Goal of the community agreement

The "Plant Sciences Community" as defined in this document, is a network of professional membership organizations that represents plant biologists focused on the advancement of science for the benefit of society. The goal of this community agreement is to present a collective vision for how we, the Plant Sciences community, want to define our values and set expectations and measures for accountability for ourselves, our staff, and all affiliated participants of our collective gathering.

II. Community Values

[CONFERENCE NAME] strives to ensure the full participation, safety, and support of all participants. We work to uphold inclusive and welcoming antiracist and decolonizing practices and values; to center and support queer, trans, non-binary, and gender minority scientists; to provide equitable access for scientists with disabilities; to invite and uplift contributions by scientists of all religions, belief systems, and cultural backgrounds; to elevate the work of researchers in underserved communities and universities; to strive for more equitable access to resources in those communities; and to open doors for those whom science has historically excluded.

III. Conference Participant Agreement

As a participant in [CONFERENCE NAME], I agree to do my part in building a welcoming and inclusive conference environment by:

- Upholding and holding others accountable to the above-described community values while attending formal events as well as unofficial activities while at the conference.
- Critically evaluating implicit biases and reflecting on how they impact our interactions.
- Treating others with civility and actively working to avoid, prevent, stop harassment, discrimination, and behavior that is threatening, intolerant, abusive or violent.
- Upholding the highest standards of scientific and academic integrity during the conference, recognizing past and present contributors to science, while

acknowledging that science can be flawed and historically has harmed marginalized communities.

- Evaluating the work of colleagues equitably—with open-minded fairness and respect.
- Encouraging and promoting climates where multiple scientific perspectives may be freely expressed and valued.

This agreement was developed by a group of plant scientists as part of the NSF-funded Root & Shoot Research Coordination Network (NSF RCN LEAPS #2134321). By attending [CONFERENCE NAME], you agree to abide and uphold this "living" community agreement in its current form while contributing to amendments in the future.

Appendices and links to reporting documents

A: Justification for community agreement

B: Desired Outcomes and Unacceptable Behaviors

C: Information on reporting: FirstDraft_GroupB

D: Additional References

Reporting Structure

Inclusive Conferences Working Group B

12/12/2022
Shawn Abrahams, Morgan Gostel, Theo Newbold, Kanwardeep Rawale, and Andrew Read, with input from Roger Innes

> Conferences are important places to learn, share cutting-edge research, and build a sense of community. Several challenges have prevented the inclusion of the entire scientific community and equitable treatment and access for those able to attend. These challenges manifest in a range of ways, from logistical (financial support, travel, caregiver responsibilities, lack of accessible and safe accommodations, etc.) to cultural (trainer-trainee power dynamics, stereotypes, "alcohol culture," racial and gender bias, etc.). In holding meetings that combat these challenges head-on, it is essential to communicate expectations to meeting organizers and to ensure safe and robust mechanisms for reporting and responding to infractions.

Purpose: Our group believes that an effective reporting structure holds scientific organizing committees, meeting attendees, and adjacent people (contractors, staff, etc.) responsible before, during, and after meetings. Here we communicate our thoughts on how to begin to implement reporting structures at each of these levels.

Introducing new terminology: This document introduces the term Interpersonal Conflict and Violence (ICV) as an umbrella term for citations of direct violence such as assault, sexual misconduct, or rape, as well as non physical acts of violence such as the use of slurs, racialised, ableist, or homophobic assumptions, microaggressions, sexist, ableist, homophobic, transphobic, anti-indiginous, or anti-semitic language, inappropriate or harmful gestures, etc. This term is meant to simplify language around instances of misconduct and should not be seen as an attempt to minimize individual persons experiences with racism, sexism, ableism, homophobia, transphobia, or other centered acts of hate. In the current document this term is not fully used until the final section, however we believe it could be integrated more fully into this document and, potentially, used in other places within Root and Shoot.

Key Points

Pre-Conference Reporting and Feedback

- Decide if it is appropriate and within the scope of the project to create meeting rating metrics – if so, post these in a centrally located place on the Root and Shoot project website

Reporting and Feedback During a Conference

- Contract with an ombuds-person and/or mediator to participate in conferences to uphold community agreements, facilitate reporting, provide training for community support volunteers, and provide a means for conflict resolution.
- Implement rapid, anonymous reporting mechanisms on-site at conferences either through synchronized push notifications via the conference app, feedback kiosks, “how are we doing” stanchions, and/or an “ideas board” that allows participants to post sticky notes that highlight key moments from their conference attendance.
- Compare and track metrics from these reporting structures both within and across societies; ensure that reporting structures are consistent across conferences so that outcomes can be aggregated and compared.

Post-Conference Reporting and Feedback

- Contract ombuds staff or work with an external service provider
- Form a committee or board whose job it is to implement and oversee ICV reporting
- Draft and implement a plan for directly addressing and resolving reports of ICV
- Create an ICV specific post conference survey based on the society's needs and specific objectives.
- Follow up on reports of ICV and center reporter needs and experiences

Body of Group B Report

1. Pre-Conference Reporting and Feedback

Although outside of the original scope of this working group, there is an opportunity for the multi-society Root and Shoot project to create a system to report on the resources, safety, and accessibility of each society meeting. As potential meeting attendees and mentors of attendees, it would be helpful to have a one-stop place to determine if a conference is satisfying our requirements for a safe and engaging meeting. We recommend that these reports would be gathered concisely on a page on the Root and Shoot site. We recognize that a promise of support and accommodations is not a guarantee, and some post-conference scoring will need to be implemented. Implementing this structure will be challenging, but is possible and worth the investment. We suggest that a volunteer committee chaired by a Root and Shoot steering committee member take on the work to maintain and publicize this resource.

We propose a scoring system for each society meeting that includes aspects such as;

- Does the society have a community agreement (or equivalent) that describes how they resolve infractions?
- Has the society committed to balancing speakers based on: career stage, gender, and geography?

- Will the meeting offer support for those with additional audio or visual needs? (e.g., ASL translator, closed captioning, slides available)
- Does the meeting offer support for parents or those with caregiving obligations? (e.g., lactation room, family activities, daycare, quiet space)
- Are hybrid options available? If so, are they engaging (beyond simply passive viewing for remote)
- Is the city/area relatively safe for our most vulnerable members – nowhere is 100% safe for anyone, but there could be some metrics/ratings available (e.g., history of police brutality)
- Has society delivered on past promises? This would be blank in year one, but this is where the post-meeting surveys could be integrated. (See section 3) (e.g., addressing reports of microaggressions and blatant hate speech)

The scorecard should include;

- A comprehensive list of each conference hosted by Root and Shoot member societies
- An infographic showing services and resources
- Historical ratings of previous meetings should be accessible

The team specifically liked the idea of integrating the root and shoot logo and the NSF logo by overlaying the circular NSF "gear" border around the Root and Shoot logo - with each gear representing one of the areas of focus and filled in if the society has satisfactorily fulfilled that area. We suggest alternating red, orange, yellow, green, blue, indigo, and violet colors around the logo.

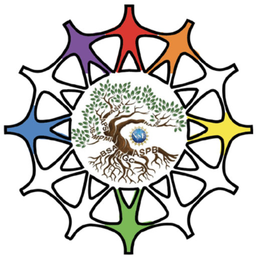

Figure 1: Mock-up of a potential color-based visual meeting ranking - here each gear represents a different accommodation provided by the meeting (red = the meeting has a Root and Shoot approved community agreement, Orange = gender balance for invited speakers, etc)

2. Reporting and Feedback During a Conference

An essential component of reporting is a real-time reaction to gauge the sentiment of attendees and provide an opportunity for conference participants to give feedback. We see similar real-time assessments of user community feedback in person, via kiosks at airport check-in booths, or placed next to restroom exits with emojis that

ask visitors to "tell us how we're doing" by pressing the appropriate emoji button. We see increased "real-time" assessment and reporting virtually, whether a brief 2-question post-phone call questionnaire or a 3-question pop-up survey after completing a webpage form. These reporting mechanisms are convenient, quick, and often anonymous. Most conferences now use mobile phone applications to organize conference programs and events; an in-app real-time reporting feature should be incorporated to provide conference participants with a rapid and convenient way to report both positive and negative experiences as well as violations of the community agreement. Reporting of this type might be ideal for assessing large-scale, overall impressions; however, the experience of conference participants may be much more direct and interpersonal and may involve severe violations of community agreements or even local laws.

Reporting complex interpersonal experiences can be a challenge due to the sensitive nature of these experiences. The severe implications of interpersonal interactions also emphasize the importance of reporting, as time can be critical for addressing and resolving conflict, recognizing interpersonal dignity and respect, and the sensitive nature of legal requirements. Regardless of potential disputes that may arise during conferences, a private and safe place to report them is critical to protecting participant needs and upholding community agreements. We propose the Root and Shoot community advocate for an independent ombudsperson and mediator to be present during conferences to provide a space for attendees to come forward with issues specific to the community agreement that may require conflict resolution and mediation. This ombudsperson would be introduced at the conference's opening and be located on-site in an accessible and safe place for participants to approach them, similar to a presenter’s ready room. Preconference, the ombudsperson person could work with conference organizers to provide recommendations for program changes should they be necessary. The added benefits of this position might include hosting training for community support volunteers and facilitating more extensive meeting discussions.

We don't mean to dwell on the negative aspects of reporting, and it's important to highlight other elements of reporting that might facilitate collaboration, networking, and different desired participant outcomes from conferences. Reporting can also provide a mechanism to exchange ideas and provide credit for intellectual breakthroughs. Conferences might offer a comfortable space for networking and interaction among community members where ideas spark and take shape. Conferences are often where new collaborations begin, new networks and initiatives form, and researchers at all career stages experience "aha! moments". Real-time reporting can encourage participants to share these experiences by submitting notes anonymously or posting them to an evolving "ideas" board that provides a space for participants to share key moments and experiences from their conference participation. In this way, real-time reporting can uphold and strengthen community agreements by celebrating and highlighting the positive interactions that emerge during conferences.

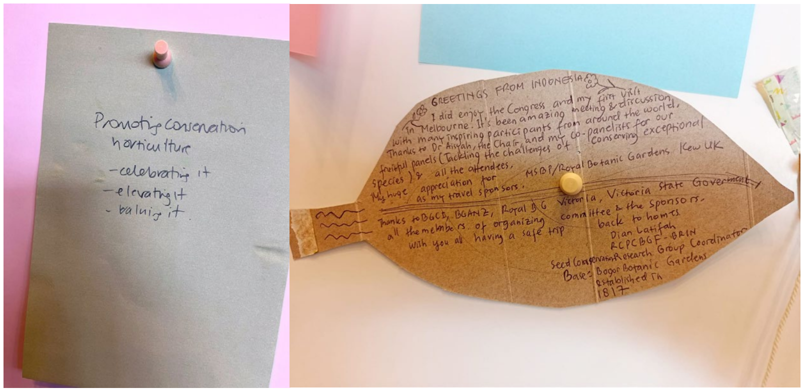

Fig. 2. Photos from an “ideas board” from a recent conference.

1. A straightforward process for reporting violations: All societies should have a reporting system available during and after the conference. We recommend a standardized community agreement violation reporting form be adopted (see Appendix). Organizers should provide an information pamphlet and/or other physical items (lanyards, etc) listing available resources and how participants can report community agreement violations. The website of every society or conference should identify a person trained in handling complaints to whom the complaints could be directed through email. The post-conference survey (See Section 3) should also contain a link, allowing different individuals to report personal experiences or potential violations. During the conference, the reception desk should have a dedicated officer to whom various participants can reach out. Wherever possible, the anonymity of the reporter should be maintained. The community agreement should encourage bystanders to report violations they witness or experience, regardless of perceived intensity or significance.

2. Community agreement violations: Misconduct/community agreement violations should be explicitly defined, and the information should be made readily available on the website of every plant biology society, which is part of the root and shoot program. Additionally, consequences for violating the community agreement should be laid out. The society’s website should include the name and contact information of the member of the society who will be responsible for the initial evaluation of the violations. The person should be adequately trained in crisis or complaint management and respond to them promptly. Within 30 days, an initial review of the complaint should be completed, after which the next course of action should be decided. Based on the severity of the complaint, either the responsible person should conclude or pass the complaint to a standing committee. Each society can develop an internal ranking or rating system, which allows them to categorize different complaints. During the initial stages of implementing the new community agreement, a third-party review of the complaint management should be performed to boost confidence in the society

members/participants.

3. Standing committee in plant biology societies: A standing committee should be created to address the misconduct/community agreement violations that occur at each conference. Initial evaluation of the complaints should be done by the dedicated officers of the society that have been adequately trained in handling complaints. No information should be made public during the time a complaint is being evaluated. If the severity of the situation requires further action, a committee of at least five members should be developed, which should include a trained officer from the society, a president-representative from the society, two institutional representatives (one representative each from the institution, where both the accused and the accuser work), and an independent third-party member (Ombudsperson). Different committee members should be asked to provide a conflict of interest document (something very similar to federal grants) to avoid any conflict of interest. The committee should be responsible for conducting a fair and timely evaluation of the complaint, and an initial report should be prepared within 60 days of the committee's formulation. Once a resolution or conclusion has been reached regarding a complaint, no public release should be done without contacting the people involved in the complaint.
4. Bystander intervention: Each society should have a bystander intervention program that encourages individuals witnessing the community agreement violations to intervene when they feel it necessary. The society should work closely with the organizers or venue staff, allowing the society to have an independent eye on potential violations. The society could conduct a seminar where representatives of the organizer or venue staff are briefed about the society's community agreement and what potential behaviors are considered violations. It was suggested that session organizers should do an online bystander training before the conference.

5. Consequences: It is important to highlight the consequences of community agreement violations. Per our discussions, different violations ranging from identity-based discrimination to sexual harassment should be dealt with severely, and consequences should be developed for each case after sincere discussion and evaluation. Potential consequences could be a) warning, b) removal from the meeting without refund, c) prohibition from attending future meetings, d) reported to their employer, e) training, f) removal from society membership, and g) reporting to law enforcement when appropriate.

3. Post-Conference Reporting and Feedback

A critical component in gauging conference attendee experiences and satisfaction is post conference feedback. This feedback is often used by conference organizers to determine how well a conference went, how engaged and active participants were, and what participants want to see in the future. These surveys are also an opportunity for participants to give constructive feedback on negative experiences.

Unfortunately current post conference survey structures are not equipped to support feedback on issues of interpersonal conflict and violence (ICV) for several reasons.

1. Attendees often complete post conference surveys months after a conference has ended. By this time any incident of significance has been shared with a supervisor, friend or colleague, or with the society's DEI committee and is being handled either informally or not addressed at all. At this point reporting an incident formally through the survey would likely be unhelpful for the person who experienced the conflict since it occurred so long ago.

2. Post conference surveys are often too general and impersonal, or too long to support sharing experiences of ICV. Either surveys generally ask how much people enjoyed conference events on a rating scale or through arbitrary categories like good, better, best and leave a text box at the end for additional comments, or they are exhaustive and leave little time to provide ICV specific feedback.

3. There is no formal structure in place that allows societies to effectively and safely follow-up on reports of ICV via these surveys. If someone reports an act of discrimination, hate speach, or prejudice there are few if any society policies that instruct staff or leadership on how to follow up on these incidents leading to people to (a.) feel like they are sharing traumatic information into a void and are unlikely to report in the first place, (b.) worry that their experience will become a subject of gossip among the society or be shared with the transgresser, or (c.) leave the society because they feel the society does not take incidents of ICV seriously.

How then can societies use post conference surveys to support their members and assure that members feel comfortable enough to report?

1. Develop a survey that is sent out to participants one week after the conference which directly addresses ICV. This will reach attendees shortly after the conference and give them the ability to report promptly. This will also show attendees that the issue of ICV is separate from conference satisfaction and event planning. We recognize the challenge of establishing and maintaining a group to objectively review the survey, but believe that with the support of Root and Shoot, it is possible.

2. Develop a clear and concise plan for addressing reports of ICV with staff and leadership that directly serve to support reporters and can connect them with an ombuds-person or the DEI committee to support society wide change in appropriate instances. The process of reporting and follow up should be transparent and shared in the post conference survey so that reporters know they will receive support.

    i. Surveys would be reviewed by a board or committee that encompasses society leadership, staff, and general members.

1. If funding is available this team of reviewers would be composed of Root and Shoot volunteers from other societies. This would assure a degree of anonymity as well as reduce possible bias from people who participate within the society group.

ii. Surveys would be collected and reviewed, situations that require immediate assistance such as issues of sexual violence, or racism and discrimination can be prioritised. With each committee member taking on a subset of surveys.

iii. Committee members will work with the ombuds-person or an external program to prepare a plan for addressing the incident. Should this require legal involvement the committee member would be responsible for reaching out to the reporter to determine if they would like to press formal charges and help them do so with the local authorities.

iv. If incidents do not require legal action a plan for addressing the incident will be drafted and shared with the reporter. The committee will work with the reporter to reach a satisfactory resolution. Resolutions could include consequences such as those listed in the "During Conference" section of this document and beyond.

By optimizing the post conference survey experience and assuring a formal structure for addressing issues of ICV we believe that attendees will be more likely to report incidents that arise during major social gatherings, and that professional societies will be able to address these issues effectively.

As part of this post-conference reporting structure we recommend that each professional society seek to optimize their reporting scheme according to the needs of their organization using a suggestion based survey and by creating a society specific task force for implementing these new structures such as the committee described above.

Appendix

Standardized community agreement reporting form:

It would be helpful for the societies involved with Root and Shoot to develop a unified form for reporting macro and micro-aggressions. We include a draft of what this may look like, but invite the leadership to consult with experts and improve upon this. We feel it is particularly important to include some version of the question, "what would you like done", as there are times that people will want follow-up action and times where just bringing the incident to the attention of the conference or Root and Shoot is enough.

Are you a participant at CONFERENCE NAME?

Is the person for whom this report is about a participant at the CONFERENCE NAME?

Date of reported incident:

Location of reported incident (e.g. within the convention center, hotel, or off site):

Description of the incident and context (please provide as many details as possible):

Were you directly involved in the incident or indirectly involved (observed or heard about via second hand knowledge)?

Offending individual’s name (or description if name is not known or you wish not to disclose):

Did you or anyone else ask the offending individual to stop or address their behavior directly?

Desired outcome or resolution:

What is your name and contact information so that we can follow up with you regarding this incident?

Name:

Email:

Phone number:

Transparent Site Selection

Inclusive Conferences Working Group C

Overview

Transparency in conference site selection is essential for society members to understand the elements of equity and inclusion being considered when a choice of site is made. There are undoubtedly essential aspects of organizing a conference that must be considered to keep costs low for attendees and to allow the conference to be successfully held, such as venue price and size and level of local support by society members to host the conference. However, participating membership is diverse and their experience must be considered when choosing the conference locations. Dissemination of some specific criteria used to select a conference site should be conveyed to society membership on an annual basis.

The overall recommendations being developed for this document are dense and provide an in-depth set of criteria for site choice. Here we provide an abbreviated set of topical information that societies will use to convey the level to which the broad criteria in this document were considered in site choice. Our goal is to have a cross-section of factors that will communicate inclusion in a one-page statement to be posted on the conference site and in the conference App. This is intended to be evidenced based for each site choice annually and **supplemented with links to local information** to make it easy for society members to decide for themselves how inclusive the site will be for them. Under five primary topics we provide points to emphasize within this communication.

Overarching

Who were the members of the site selection committee?
Were multiple sites considered? Or just one put forward?

Sustenance

Did organizers perform an initial screening of food options within and surrounding the conference site and lodging? Do options reflect the diet diversity of attendees (vegan, vegetarian, gluten-free, etc)?

What is per diem for the host city, and do local food establishments have options that fit within the per diem budget?

Are food vendors available at all reasonable hours? Are there any seasonal changes in business hours that might affect conference attendees?

Location setting

Does the surrounding population reflect the diversity of conference attendees?

Have local or state governments enacted laws that may endanger attendees based on their identity?

Are there people available that can provide on-the-ground organization and recommendations?

Are there sufficient food options nearby that can accommodate a variety of dietary needs?

Are there affordable, nearby lodging options that can accommodate diverse lodging needs (e.g., affordable single rooms or shared rooms only)?

Have you provided mechanisms for attendees to easily and safely return to accommodations if events go late into the evening?

Have you built in enough time, and does the venue have enough space for non-overlapping social events?

Venue

The specific building or building complex in which the majority of the conference will be held

Is there any known ownership group history or current practices that have led to worker disputes in relation to equitable treatment of staff and does the venue adhere to fair hiring practices.

Does the size of the venue not only consider space for professional presentations and meetings to accommodate the society membership, but also provide space/rooms that can be utilized for special needs, such as quiet rooms, lactation rooms.

What criteria were used for selection of volunteers (was diversity considered) at the venue and how will they be compensated for their time (meals, registration, free time) and will accommodations be made for their attendance at talks, etc.

Does the venue comply with Americans with Disabilities Act (ADA) accessibility standards? What provisions are there in conference and meeting rooms and poster session space (e.g. wheelchair accessibility)?

What day care accommodations are available at the venue?

Have you considered the community local to the potential conference location and invited community members to attend so they can both benefit and contribute?

Transportation

Is there an international airport in the area accessible by budget-friendly airlines?

Are there other affordable options than air transportation for reaching the site (e.g., train, buses)?

Is there public transport to move to/from the conference site and around the city? If yes, Are you providing free daily passes for public transport? Which are the criteria for obtaining these free passes?

Conference Accessibility

Inclusive Conferences Working Group D

Patricia Baldrich	patriciabaldrich@gmail.com	Donald Danforth Plant Science Center
Adriana Hernandez	aherbotany@gmail.com	Cal Academy of Sciences
Imeña Valdes	imenavaldes2020@u.northwestern.edu	Northwestern University/Chicago Botanic Garden
Joanna Friesner	jfriesner@danforthcenter.org	Danforth Plant Science Center & NAASC
Melanie Link-Perez	mlinkperez@gmail.com	The University of Tampa
Sarah Boggess*	sbogges1@vols.utk.edu	University of Tennessee

*added by Denita Hadziabdic-Guerrydhadziab@utk.edu, University of Tennessee

We suggest starting or at least cross-referencing with a previously published guide:
https://www.sigaccess.org/welcome-to-sigaccess/resources/accessible-conference-guide/
https://www.adainfo.org/hospitality/accessible-meetings-events-conferences-guide/

Lead/co-lead(s):

1. Physical environment: Melanie/ Adriana
2. Audiovisual environment: Imeña/Pat Melanie
3. Family environment: Pat/ Joanna
4. Economic environment: Joanna/ Imeña
5. Wellness environment: Adriana/ Melanie

INTRODUCTION

Accessibility is the foundation of an inclusive conference. In addition to its social justice aspect, accessibility is a fundamental requirement; for example, 1 in 4 people in the United States have a disability according to the CDC (https://www.cdc.gov/ncbddd/disabilityandhealth/infographic-disability-impacts-all.html). Ideally, professional societies will choose conference locations that will already have in place many of the suggestions below; however, the reality is that some sites are unable to provide full accessibility options. In time, conference sites may increase their accessibility options as conference organizers request and demand accessibility features. **Indeed, inclusive societies have an obligation to use their influence to continually push for increased accessibility. Accessibility should be part of the conference planning from the start: site selection choice, budget development, and program structure. Inclusive societies have accessibility as a core value.**

The best accessibility plans will fail to achieve their objectives if they aren't clearly communicated, in advance, to communities. **What is outlined in this document is offered as 'basic best practice' accessibility features, regardless of whether they are requested or not. Organizations should survey their community (speakers/presenters and prospective participants) during the conference development phase to gain input on their priorities to help guide decisions. Then, include several questions during the registration process to determine what types of accommodations are needed.** These questions will enable the organizers to provide additional accommodations as needed. Conferences should establish communication mechanisms (e.g., email address, Google form, Slack, etc., that are monitored and responsive) and invite the community to give input as early as possible with their accessibility needs or concerns. Make clear that you are committed to enabling an inclusive environment and broad participation, and then follow-through on that commitment.

This section is divided into five accessibility "environments": Physical, Audio/Visual, Family, Economic, and Wellness. It includes recommendations and check-lists to enable broad and inclusive planning. Conference websites should include an "accessibility" section, visible on the main menu so users have just one click to locate it, that makes clear the accessibility features you will provide and invites community contact to resolve potential questions and issues. When the recommendations in this document cannot be met, clearly indicate relevant information on the accessibility page so that participants can plan accordingly. Some participants may need to make individual arrangements, some of which should be financially supported by the conference through an "accessibility" or "equity" fund. While dedicated fundraising for accessibility is recommended, a complementary approach is to designate a percent of each registration fee for the "equity fund". It isn't always straightforward to determine the level of equity fundraising a society should pursue; some things are easier to quantify (e.g., 25 awards of $500 each for early career participants), whereas the accessibility needs of individuals may vary. Surveying for community input, prior to and during conference development, may help guide appropriate budget planning. Ultimately, decisions may need to be made based on limited resources and societies that engage their members in this discussion will better serve their community. Ideally, such discussions will raise awareness, and, by tapping into collective community creativity, develop new solutions.

(1) ECONOMIC ENVIRONMENT

Participating in scientific conferences is an expected part of being an academic scientist and considered vital especially to those earlier in their career, including students, postdoctoral scholars, and new faculty. There are other groups that also particularly benefit from the networking and collaborative opportunities at conferences, such as faculty at primarily

undergraduate institutions (PUI) and members of groups that are under-represented in STEM. Paradoxically, these groups tend to be those with the fewest resources; thus, the gap between the well-resourced and resource-poor is exacerbated unless inclusive conference approaches are employed. Early in the conference planning process, societies should develop approaches and options to enable broader participation, with a significant priority given to those with the greatest financial need. Starting as early as the initial budgeting and site selection process, each step should consider how options may affect broad participation. This is a "triage" opportunity; in other words, consider how the budget affects those with the most, or the least, financial need.

A common practice has been to extend financial support to invited speakers who tend to be mid- or late-career, well-established, and often tenured, faculty; however, they tend to be the most-resourced community members. It is recommended to develop a budget that prioritizes early career scholars, participants from less-resourced institutions, and/or attendees from less wealthy nations. For example, you may offer 'more-resourced' invited speakers complimentary registration and reserve additional budget for support of less-resourced invited speakers and other similar participants. The conference can be structured using various approaches so that those with the least resources are prioritized for discounts, or for direct funding, to enable their participation. Communicating your priorities early to your community will convey your society's inclusive values and, if the revised budget is a significant shift to your standard operations, may clear up confusion. For example, if your society revises the budget such that invited speakers will be offered reduced benefits from previous norms, conveying this change, and the rationale behind it, provides transparency. The new budget approach should be conveyed explicitly in speaker invitations. One possible approach is to offer invited speakers a standard benefit, and give them the option to opt-in to a previously-extended benefit. In other words, the invitation could extend free registration (a standard benefit), but make an additional benefit (e.g., travel subsidy) by opt-in request. It should be made clear that their choice will remain confidential, and if the speaker is in need of the travel subsidy, that they can easily opt-in to receive it by stating "I request the travel subsidy" when they reply to accept the invitation. These conversations would remain private between the society and the invited speaker, and thus, speakers shouldn't feel undue pressure to decline funds they truly need.

1. Registration fees should be tiered and subsidized for less-resourced participants
    a. **Discounts or subsidies for participants from developing countries**, if an international conference. Investigate the relative funding rates of students and faculty in countries or regions that may want to attend. Consider offering discounted rates to attendees from such regions (e.g., the Global South). Raise unrestricted funds to support such attendees (i.e., defrayed or free, registration).
    b. **Set fees according to career level**, charging early-career participants less than later-career participants. Those with more resources subsidize some of the cost of lower-resourced individuals (e.g., students rates vs higher faculty & industry rates).

Keep in mind additional categories that fall between the lower student & postdoc tiers and higher tenure-track faculty tier, such as research scientists, non-profit staff, and teachers/faculty from Primarily Undergraduate Institutions (PUIs).

c. **Consider the cost/benefit of everything that goes into the registration fee cost**. If the conference includes activities, charging the cost evenly in registration fees may make the fee too expensive for many. If the activity is ticketed separately, the charge is borne by those with resources; however, this generates a divide between the well-resourced and the less-resource attendees. Some approaches: tiered level of activity fee; fundraising to subsidize activity costs, combined with tiered pricing; fundraising for attendees that request a fee waiver or reduced ticket price; and develop less expensive activities, to be more inclusive. These approaches apply to all costs, e.g., management services, catering, and facilities.
d. **Offer a reduced (or waived) rate for a caregiver for a disabled participant who needs to bring a companion/aid with them.** The fee could be waived entirely, if possible, or could be limited to some core costs such as meals, coffee breaks, or staff management fees.
e. **Consider adding a partial-meeting fee for participants that can only attend several days.** Some participants are only able to attend a conference for a day or two due to family obligations, personal disability or illness, or other responsibilities. While the success of conferences, including financially, relies on full participation by most people, allowing a subset of participants to opt-in to a single or multiple day fee could expand participation.

2. Conference participation awards should prioritize those with fewer resources and support the objective to diversify participation.
   a. **Offer grants for underrepresented communities with financial need**, e.g., members of underrepresented groups by race or ethnicity, gender, sexual orientation, gender identity, historically under-served and under-resourced institutions (e.g., Primarily Undergraduate Institutions, public regional universities, or HBCUs), first generation to attend college, etc., collectively “minoritized” participants. Members of underrepresented groups or under-resourced institutions are less likely to have the resources to participate fully in conferences. The application should make clear that these are enabling awards to expand inclusion to members of underrepresented groups that lack the resources to participate without the funds.
   b. **Offer grants to early career scholars with financial need,** e.g., students, postdoctoral scholars, and pre-tenure faculty. The application should make clear that these are enabling awards to expand inclusion to those early career scholars that lack the resources to participate. Since some institutions have pots of funding dedicated to early career conference participation, consider asking applicants to list any additional sources of funding they have (e.g., department travel award). For faculty (e.g., if you include pre-tenure faculty), you can request they list their ‘current

& pending support'. Include optional space for them to address their support levels, e.g., if the faculty member has a large lab, travel funds may be spread thin and thus, funding is needed. Or, the faculty member may wish to attend your conference that is out of their 'core research area', and thus, seek cross-field funding. These data can be used in the selection process to determine where limited funds are allocated.

c. **Include a "financial need" funding category** with unrestricted funds. Some participants will not qualify under standard award categories due to, for example, career stage or citizenship status. Consider offering funding possibilities to those that express financial need, e.g., a partial or full registration waiver. Societies will need to determine eligibility. See 'inclusive fundraising'.

d. **Avoid offering awards only as reimbursements**, especially for those participants that are early-career, minoritized, and/or from a developing country, PUI, or HBCU/1890. Offer options that ensure recipients aren't out of pocket for expenses, especially if they must wait weeks or months for reimbursement (i.e., directly defray fees or lodging). Consider paying directly for travel costs (i.e., booking flights). Give awardees the option to choose from possible ways to receive the awards (e.g., waived registration, travel costs, lodging).

3. Inclusive Fundraising

a. **Solicit unrestricted funds**. Conference awards are often derived from restricted sources such as government grants that can be used only for specific purposes, e.g., travel costs for specific speakers, or early career participants with specific citizenship. Unrestricted funds enable broader inclusion; these are funds that aren't earmarked for specific purposes or people. Societies can use unrestricted funds to offer "financial need" awards for those that don't otherwise qualify for funding. Funding could be further organized into, e.g., "early career", "global South", "parents of young children", or "general financial need" support. Unrestricted funds are commonly provided by commercial or non-governmental sources such as journals, industry groups, or societies that are relevant to your community and you have relationships with.

b. **Develop a short fundraising 'pitch' that includes the conference's draft program, your 'ask' (e.g., sponsorship), and what, if anything, the sponsor will receive** (e.g., named as a session sponsor; inclusion of their advertising flier in materials). It's often effective for fundraising requests to come from a personal connection (e.g., an organizer who is a journal editor or reviewer could contact journal staff or leadership directly.) Make requests at least 6-12 months before the conference; many groups commit their budgets on an annual basis.

c. **Establish an "Accessibility Fund" to enable inclusive participation**. If your conference supplies transportation (e.g., shuttles from lodging to conference site), the best practice is to ensure broad accessibility, such as wheelchair accessibility. If this is not possible, make clear on the conference accessibility webpage that

provided transportation lacks wheelchair accessibility and participants with additional needs can apply for accessibility funds (e.g. Uber, taxi). Make the application simple.

   d. **Consider adding a small optional fee during registration for attendees to support inclusive participation**. Such an ‘equity/accessibility fund’ could be used to support participation for those with financial need or don’t qualify for other sources. Ensure that the optional added fee is clearly marked as a separate ‘donation to equity/accessibility fund’ on the receipt, as it may not be reimbursable by certain funding sources. Consider exploring alternate ways to receive donations so that they’re clearly separated from conference costs (e.g., Venmo, PayPal, and the like).

4. Conference lodging and travel
   a. **Establish sufficient lower cost budget housing options**. Consider contracting with local universities that offer inexpensive summer student housing, if relevant to your meeting dates. Make sure the housing is accessible and provides adequate amenities (e.g., proper bed for those with physical disabilities, microwave/refrigerator for those on a budget, with a need to store medicine, and/or with specific eating habits, etc.)
   b. **Seek a range of low- to mid-priced hotel options that have decent ratings**. Consider arranging room blocks and discounts by contacting hotels for meeting discount codes. Ensure the block contains different types of accessible rooms (e.g., roll-in shower, hearing impaired.)
   c. **Describe clearly on your website the various pricing levels of lodging**, e.g., budget, moderate, higher end. Include important information such as: distance from the venue, proximity to public transportation; estimated time to walk to the venue; taxi, Uber, Lyft, etc. availability and estimated costs for traveling to the conference venue.
   d. **Provide “roommate matching” for attendees that book lodging via the conference portal**. This can occur during the online registration process where attendees may opt-in to share a room with two beds, typically with someone of the same gender. This approach will likely require management. A less hands-on approach is to set up a communication system to allow attendees to seek roommates and manage their own arrangements. This approach comes with some risk as the individuals are unknown to each other, and relationships and finances are managed by them.
   e. **Accessible transportation:** Ensure that conference lodging is accessible by public transportation and/or within walking distance of the main venue. Provide “transportation matching” for to/from the airport, particularly if public transportation is less available or reliable. This could be arranged in advance of the meeting to coordinate arrival at the nearest local airport (as applicable) where participants meet at a designated spot at the airport arrival, and take a taxi/Uber/Lyft to the conference

site or the common lodging site. This could be arranged informally or formally at the conference for the final day (e.g., set up an easel with times for people to sign up and travel together.) Set the expectations and guidelines clearly as to how payments are to be handled, particularly if it is to be done by the participants themselves (without organizer mediation.)

f. **Volunteers to help at the conference in exchange for funding support.** Consider if there are appropriate conference tasks that could be taken on by some attendees in exchange for free or reduced conference fees. For example, local students, postdocs, or staff may know the area well enough to serve as on-site guides before, or between, sessions. Or, helping pass around microphones during the Q&A sessions; distributing materials at the registration desk; or assembling conference materials before the meeting starts. Ideally, helpers aren't required to work during sessions so that they can participate fully, opportunities are spread across labs, and expectations and compensation should be transparent at the point of invitation. Additionally, the work hours should be reasonable for the amount of funding provided. A best practice is to develop a 'contract' outlining the expectations of the volunteer and the organizing committee (i.e., what the volunteer is committing to do, and what they receive in exchange, and the consequences if either party doesn't fulfill their obligations). This contract should be agreed upon in advance, and signed by both the organizer and the volunteer, and each retains copies. Each society should determine if they want to establish an enforcement mechanism /process.

5. Food at conferences
    a. **Provide affordable options for meals not included in the conference fee.** Most conferences include at least some food within the registration fee. However, many do not fully cover meals throughout the conference, and meetings in high-cost areas present additional challenges to many participants. Provide a map, list, and recommendations for a variety of lower cost restaurants and grocery stores within walking distance of the venue, especially if meals during the conference are not included and participants are expected to fund themselves.
    b. Make clear on the conference website well in advance the meals to be included in the registration fee and those that are the responsibility of participants. Link this information to the list of lower-priced stores and restaurants that you developed.
6. Hybrid option
    a. Conference participation is expensive, especially for attendees that must travel to the site and stay in lodging. Beyond on-site costs are fees such as to secure travel documents, take time off from work, possibly unpaid, or arrange for caregivers for family members. Hybrid meetings have recently been widely adopted in response to the global Covid-19 pandemic and have expanded access to many people that otherwise would be unable to attend.

b. Similar to accessibility, hybrid components should be planned carefully and early and not as an afterthought. Resources (time and money) are required to provide a worthwhile virtual experience; societies likely will need to consider possible features and prioritize them. Engaging your community in transparent discussions of costs and options can help guide your choices and increase goodwill. Ideally, open discussion will increase community buy-in and perhaps creative problem-solving. Ensure that accessibility features are part of the discussion.

(2) PHYSICAL ENVIRONMENT

The physical environment of the conference includes the conference venue (where presentations and the scientific and social events will take place) as well as the conference hotel or other overnight lodgings; attendees will need to travel between and navigate these spaces during the conference, so it is imperative that organizers assess the continuum of these physical spaces to ensure accessibility.

1. Communication of accessibility arrangements
    a. Accessibility arrangements should be clearly detailed on the conference website so attendees can plan for their individual needs and assess their risks/safety.
    b. Provide an accessible conference map, including directions for drivers, cyclists, walking, and public transit, wayfinding for wheelchair accessibility including elevators, accessible bathrooms and entrances, and locations of specialized rooms, such as lactation room and quiet room. Ensure that the map itself is accessible (by a screen reader, etc.); if provided as a PDF, add alt-text.
    c. Images of main meeting rooms. These can be emailed or otherwise made available to attendees before the conference starts and/or included in the conference map. Also useful is to include this information on a conference app (for phone or tablet use), which is becoming more common.
    d. Offer a buddy system
        i. This could be a sign-up sheet to help participants match up with other attendees with disabilities, OR pay students to pair with participants who require physical help/guidance around the conference for specific events like mixers
        ii. Other ways that the buddy system could increase accessibility is through improving safety, such as escorts to/from restaurants/bars at night or pairing people for early morning workouts/running
        iii. Consider developing a way to vet potential buddies, and make it clear that all interactions between buddies still fall under the Community Agreement
    e. Provide the necessary accommodations for service animals, such as a place for the animals to relieve themselves, and contact information for a local vet.
2. Site visit

a. The local committee should walk the venue and ensure that it is accessible to those in wheelchairs and/or those with low-mobility. To ensure that there is adequate assessment, request that the venue hosts prepare, in advance of a site visit, documentation of the venue's accessibility features. Use that list as a starting point to discuss internally and with venue staff whether accessibility is addressed.
b. Prior to signing contracts, discuss with the site management the available accessibility features, including what is required by local laws (e.g., ADA compliance in the USA)
c. Appoint a local accessibility chairperson for the conference
d. Provide secure storage (for items people with disabilities may have to carry)
e. Ensure that poster session aisles are wide enough for those with low-mobility or who use wheelchairs or other mobility devices; consider making aisles "one-way" for easier navigation
f. Accessible auto and bicycle parking near the venue
g. Ensure that if there is a stage for speakers, that there is a ramp for those with mobility issues
h. Ensure adequate signage for attendees that use wheelchairs so that they can easily navigate safely from conference lodging and local transportation (if relevant) to the meeting site. Include signage within the conference venue so that attendees can easily find elevators or other assistance.
i. Examine the lactation room and ensure that it meets the basic requirements (outlined in Family Environment below)

3. Two quiet/low stimulation rooms (for those with "invisible wounds", such as survivors of trauma, individuals with neurological conditions, and anyone who needs a break). One room is for quiet & calm, the second (if needed) for participants that may need to have accommodation for potentially disruptive behaviors (physical or verbal outbursts)
4. Accessible seating clearly labeled along aisles in every room with seating
   a. Furniture with and without arms
   b. Chairs that aren't connected to other chairs so seat placement can be adjusted when needed.
   c. Furniture for people of various sizes
   d. Be able to reserve aisle seating for people who may need an easy exit
   e. Aisles left clear and at least 36" wide for wheelchair accessibility
   f. Priority seating (labeled) at front of rooms for individuals who are hard-of-hearing, have low-vision, are deaf and need to be near interpreters, and so forth
   g. Some rows with open spaces without chairs where a wheelchair could go, rather than having to move chairs or put the wheelchair in an aisle

(3) AUDIO/VISUAL ENVIRONMENT

Worldwide, 8.5% of people have some form of hearing loss and/or vision impairment. In the United States, 30% of working professionals have an audio/visual disability (20% are deaf or hard of hearing and 10% have some degree of vision loss) (https://www.3playmedia.com/accessibility-online-video-stats/). This is a considerable number of people who require some type of accommodation in order to benefit fully from conference participation. Organizers should survey their community well in advance to determine accessibility needs that should be addressed. For conferences to be accessible to this population, we provide various ways to accommodate different degrees and types of audio/visual needs for both in-person and remote attendance.

1. The conference website should follow the W3C Recommendation from the Web Content Accessibility Guidelines (WCAG) 2.0 and be tested for functionality before sharing (https://www.w3.org/TR/WCAG20/).
2. Several microphones should be available in every conference room. Additionally, we recommend providing headsets that link directly into the A/V system. Survey participants during registration so that enough headsets are made available.
3. Consider providing sign language services on-site (and include good lighting so the audience can see the person signing) or remotely. We recommend asking participants about their needs during the registration process so organizers know in advance the accommodations that are needed, and can address them by sourcing local interpreting agencies ahead of time in order to better anticipate costs.
4. Live caption writer on site and/or remotely.
5. CART captioning (in the room and online) (https://www.hearingloss.org/hearing-help/technology/cartcaptioning/).
6. For international conferences, we recommend providing access to the conference webpage and documentation on a variety of platforms, taking into account some countries' webpage restrictions. For example, in China, where many common online platforms are restricted, WeChat is broadly used for communication. An additional consideration would be translating essential information on local travel and accessing the conference in different languages. One possible resource is local tourist information as cities usually have information available in different languages. If it is not feasible to translate the webpage or documentation, we recommend informing conference attendees that the automatic Google translator can be used on most web pages; alternatively, they can copy and paste information into DeepL, a free online translator: https://www.deepl.com/translator.
7. Offer an online workshop several weeks to a month before the conference to provide guidelines on how to make their presentation accessible (see Appendix A). This will benefit all conference attendees. Make this information available in written and downloadable format on the conference site at the earliest stages of meeting development.

8. The conference program should be available in various formats (print, electronic, in large print, and if the need is indicated, Braille).
9. Provide an assistant to help advance slides for presenters who may have visual impairments.
10. Reserve ground floor rooms or rooms near elevators. Ideally, rooms will have Braille labeling.
11. Provide an assistant who can read posters for blind/low vision attendees and/or provide tactile maps/audio options for posters.
12. Consider a way to submit questions to speakers electronically for in-person meetings. Slido is an application that can accommodate this (https://www.slido.com/).

(4) FAMILY ENVIRONMENT

Caregivers are a major part of the workforce, and conferences get higher attendance rates when they offer accommodations to bring children along (https://doi.org/10.1073/pnas.1803153115). The so-called "baby penalty" (www.chronicle.com/article/The-Baby-Penalty/140813) can affect career development at any stage. Here we include recommendations that could help make conferences more inclusive for caregivers with children of all ages. We point to conference characteristics that would help ensure that the conference is family-friendly and inclusive to caregivers, and provide resources they need to take care of their children. Additionally, we recommend that offering a virtual meeting component could make it more accessible to caregivers who cannot or decide not to travel.

2. Ensure that a **lactation room** is available at the conference venue. The lactation room should be clearly marked and included in the conference information, and should not be simply a closet or space in a bathroom. Consider the following recommendations (See Appendix B).
    a. If a lactation room is unavailable at the venue, we recommend bringing a portable lactation space to the venue, e.g., https://www.mamava.com/all-products or https://www.nestl-space.com/. Make sure that the portable lactation space is located outside of busy areas, to provide the user with privacy and a comfortable environment.
    b. It is important that all conference organizers and staff know what a lactation room is and where it is located. Make sure the room is clearly identified and procedures for its usage (e.g., reservations) are established and communicated to attendees and staff.
    c. Some parents may not be able to schedule the use of the lactation room around all talks and events. If possible, we recommend having streaming of the talks available in the lactation room.

3. Offering options to help caregivers with **childcare** during the conference is essential. There are several options, from offering childcare on-site, to providing grants for caregivers to bring a caregiver.
    a. Some options for professional companies that offer childcare on-site: https://conferencechildcare.com or https://www.kiddiecorp.com/ or https://ahelpinghandevents.com/.
    b. For caregivers that attend the conference with children, attending every session might be impossible. Consider offering partial registration for parents with children on site might enable broader attendance. It may include registration for individual days, or morning/afternoon shifts.
    c. Even for local scientists, childcare tends to be unavailable or complicated to find during weekends. Consider scheduling conferences from Monday to Friday, during regular working hours.
    d. Childcare is harder to find and schedules are hard to maintain, especially in a foreign city or country. To take into account the dependents' jet lag and that changes in routines might make the life of caregivers harder, we suggest not to schedule major events (like plenary sessions) in the early morning or evening.
    e. Similarly, allow presenters with attending children to schedule their talks at times that would better accommodate their family obligations. You can survey invited speakers in advance to help structure the program; if the program is already established you could survey speakers to determine if there are caregivers (or speakers from disparate time zones) to help decide speaker order to best accommodate them.
4. Ensuring that the conference is **welcoming for caregivers and children** will provide a better and more inclusive experience. Here we provide recommendations to help create an inclusive environment:
    a. State in conference materials (web page and abstract book) that the conference welcomes children (in non-disruptive ways).
    b. Provide official registration badges for kids with a fun job title (like "Future scientist").
    c. Provide a room designated as a "parent lounge" where families can spend some time with their children, and interact and connect with other families.
    d. State on the conference web page and abstract book that babycaring is welcomed during the conference.
    e. Connecting with other parents in a similar situation can strengthen belonging. We encourage conference organizers to create a social group in advance for caregivers with children that are attending the conference.
    f. Networking is an essential part of any conference. To fully include caregivers in these events, we recommend including social events for families and/or events that are family friendly with respect to location and timing.

(5) WELLNESS ENVIRONMENT

Wellness can be an all-encompassing term used to describe one's physical, mental, and emotional wellbeing. In the context of developing inclusive conferences, preparations can be made to promote the safety and comfort of all participants, including those who are often excluded from conferences because they require particular arrangements and may not otherwise have the physical and/or financial ability to attend the conference. Establishing protocols for catering, social events and social conduct/interaction, COVID and other health concerns, and preferences for interaction, will encourage participation from a wider community and will benefit the community overall. When these recommendations cannot be met, clearly indicate the information on the website so participants can plan accordingly. Some participants may need to make individual arrangements, some of which should be financially supported by the conference through a "conference accessibility fund".

1. Catering
    a. Make arrangements to accommodate various dietary restrictions, and consider cross-contamination as participants may not be able to eat from buffet settings. Survey your participants during registration about their specific needs.
        i. Please see Appendix: Wellness for a list of examples
    b. Provide a complete ingredient list so attendees can assess if foods follow individual dietary restrictions.
    c. Participant mobility can be increased by clearly blocking off the food area(s) away from crowds so low-mobility participants and participants with wheelchairs do not have to go through crowds. In addition, place the food area near the entrance so that participants do not have to navigate between tables and chairs to access.
    d. Consider providing additional catered events for low-income students who rely on catered events to obtain meals during the conference.
2. Social events
    a. Host events where affinity groups can create a sense of belonging and build community such as ethnic group socials, gender identity and sexual orientation socials, family socials for those attending with children, or other groups represented in the conference population. Allow individuals or groups to host their own event and provide financial support such as for catering to encourage participation.
    b. Host at least one non-alcoholic social such as a dessert bar or ice cream social, arts and crafts social.
    c. Consider holding a "movement" event. This can be a single event such as the "Weed Stampede 5K Fun Run/Walk" at North American Arabidopsis conferences. You might also provide yoga mats in a separate quiet room and encourage participants to use them for stretching. Consider arranging for a volunteer (e.g., from the organizing committee or a participant) to lead a yoga session open to all and at no cost to participants. This could be set up in the morning prior to sessions and/or during the day if there are breaks.

3. COVID and health beyond
    a. The conference website and the on-site registration desk should include information on where to locate first aid on-site, the nearest hospital, and local drug stores.
    b. Provide plenty of good quality masks (e.g., N95s).
    c. Conference rooms should provide excellent ventilation
    d. Conference rooms should provide CO_2 monitors, and capacity limits for optimal indoor air quality are posted
    e. Provide multiple options for outdoor space (especially for eating)
    f. Provide hand sanitizer, especially around events with buffet style
4. Preferred method of communication/interaction – Provide name tags/banners for users to indicate:
    a. Shyness/socialization comfort
    b. Physical contact comfort (shake hands/no touching/hug etc.)
    c. Pronouns
    d. Stimulation level (e.g., “easily overstimulated”)
    e. Text on name tags should be large enough to be seen from 3 feet away and printed in high-contrast (e.g., black text on white paper)
    f. Consider providing different options for how to wear name tags (hanging from neck, pinned to shirt)
5. Hybrid option - offering a virtual component will enable access to the meeting for many groups such as people with families, disabilities, financial need, visa and travel concerns
    a. Provide live captioning by hiring a real-time live captioner for main events such as keynote speakers
    b. Assign a moderator for talks to monitor and repeat questions, and provide clarifications
    c. Encourage closed-captioning for all recorded and live talks
    d. Host virtual social events for off-site participants

Appendix A: Presentation Guidelines

Both in-person and online presentations

- Title on each slide to summarize main point
- Presentations, including figures, should use colors that are colorblind-friendly (https://davidmathlogic.com/colorblind/)
- Spell out acronyms (don't just verbalize them)
- Provide pdfs of the presentation beforehand (if possible)
- Video captioning that can either be transcribed by a person or edited later (automatic video captioning typically has many errors)
- Presenters should describe the slides, pictures, and videos for those with visual impairments
- Use large font (40pt for in-person meetings and at least 24pt for virtual meetings)
- Use a sans serif font (like Arial, Verdana, Helvetica)
- Provide alt text and/or image descriptions
- Limit amount of text on slides
- Speak clearly and always use a microphone
- Face the audience when speaking for those who rely on lip reading
- Use plain language
- Avoid using idioms and other phrases that are not well known to speakers of other languages and/or people with some cognitive or developmental disabilities
- Limit the number of visuals on a slide
- All graphs/charts should be described and summarized
- Give the audience time to read your slides
- Use the built in accessibility checks that Microsoft powerpoint and other presentation builders typically have
- Presentations/workshops that last longer than 45 minutes should include a break
    - If you are including breaks let the participants know ahead of time how many and approximately when they will occur. Likewise if you are not providing built in breaks, let the participants know so that they can decide to take their own as needed.

Online only

- Turn on your video and center your face so the audience can see your lips while you talk and read your facial expressions
- Minimize background noise
- Record the talk
- Blur the background of your video
- Add name and pronouns in your profile
- Use a good quality microphone

Appendix B: Lactation room characteristics

- Ensure that the designated lactation room is accessible with a wheelchair and large enough to accommodate all needs.
- Ensure that the lactation room is located in a quiet space, and avoid passage areas.
- Do not locate it in a restroom or space that can only be accessed through bathrooms, locker rooms, or similar areas; lactation rooms in restrooms do not meet the requirements of the Affordable Care Act (ACA)
- Do your best to provide a sink, so breast pump equipment can be cleaned, and install a regular faucet instead of a motion-sensing faucet, which shuts off too frequently for effective cleaning
- Offer a microwave, so breast pump equipment can be sterilized
- Make clear access to an electric outlet
- Obtain a washable, comfortable chair
- Place a small table next to the chair
- Consider using a non-carpeted floor that can be easily cleaned
- Offer paper towels and sanitizing wipes
- Arrange for the room to be placed on the building's regular cleaning schedule
- Identify the room with a lactation space sign
- Create a locked or secure area, and provide a privacy please sign
- Assure that the room is temperature-controlled, warm and comfortable
- Post a room usage schedule, or create a Google or Outlook calendar so those who use the room can view and edit
- Consider providing lockers for storing personal items like pumps, bottles, and parts.

Appendix C: Economic Accessibility

- Site selection: while developing options consider location costs: travel, lodging, food for participants from a variety of career stages, institution types, and geographic locations attending.
- Conference budget: interrogate registration fee development and consider who (speakers, organizers) are covered. Avoid pricey activities in registration fees and/or raise external funds to support them. Ticketed activities: develop tiered costs. If catering is expensive, consider reducing food provided (to lower the fee) and map out nearby, accessible, diverse, low-cost food options.
    - Registration fees: Tiered fees to reflect varying resource levels, e.g., by career stage whereby faculty contribute higher fees and subsidize students and postdocs. Also consider: participants from less-resourced countries and institutions (e.g., PUIs, Global South).
    - Offer free or discount registration fees to participants with financial need in exchange for reasonable volunteer work.
    - Develop an external (to registration revenue) fundraising plan to support those with the most need (e.g., early career, minoritized, parents with young children, those with disabilities, etc.)
    - Solicit restricted funds (e.g., government grants) to support some participants and unrestricted funds from companies, societies, and journals, for more inclusive funding.
    - Develop non-onerous funding applications that accommodate a variety of less-resourced participants. Offer funding options that do not rely on reimbursement, especially for those with fewer resources.
- Lodging: Secure a range of decent lodging options near the venue, or easily accessible by public transportation. Clearly communicate the price range, amenities, and proximity to the venue.
    - Inquire if there are local colleges or universities that may offer low-cost residence halls.
    - Facilitate ‘roommate matching’ to allow participants to share lodging costs.
- Communicate clearly your accessibility efforts:
    - Identify on conference materials the locations of easily accessible & affordable restaurants & grocery stores to obtain budget meals (for meals not supplied by the meeting).
    - Communicate clearly on the website what is included in the registration fee, e.g., meals, coffee, and what must be budgeted separately by participants.
- Develop a hybrid option that enables meaningful participation; price the hybrid fees as low as possible (you may need external fund-raising to keep fees reasonable)

Appendix D: Wellness Accessibility - Catering

- Vegan
- Vegetarian
- Diabetic (make non-sugar beverages and foods available)
- Low salt
- Allergies
    - Celiac-safe
    - Gluten-free
    - Nut-free
    - Peanut-free
    - Soy-free
    - Fish-free
    - Shellfish-free
    - Egg-free
    - Lactose-free
    - Wheat-free
- Religious dietary requirements
    - Hallal
    - Kosher
- Low sensory
- Ensure that non-caffeinated drinks are available and clearly marked
- Have water bottle filling stations available throughout venue

Speaker Selection & Equitable Programming

Inclusive Conferences Working Group E

Goals of the document

This document provides guidelines for speaker selection and equitable programming as fundamental components of planning and achieving inclusive conference events. Specific goals of the guidelines in this document include to:

1. Bring a diverse group of people together to advance scientific goals.
2. Improve representation among invited speakers, participants, and organizers (and organizing committees).
3. Build the groundwork for an inclusive atmosphere (inclusive/equitable programming) during the meeting itself and throughout the planning process.

1. Make a Commitment

Make a commitment to invite as diverse a group of speakers as possible in terms of but not limited to race, gender identity, career stage, sexual orientation, age, disability, ethnicity, immigration status, national origin, religion, and socio-economic status, and institute type. Set measurable goals for equity and inclusion. Goals should be shared with speakers and participants through conference websites or application and registration forms in order to establish accountability and foster a friendly, open, and inclusive environment.

2. Form a Diverse Organizing Committee

Form an organizing committee that is diverse. Research shows that the composition of the organizing committee is important for the representativeness of who speaks, for example, women invite more women to speak (Sardelis and Drew 2016). Consider reaching out to different kinds of institutions (e.g. Minority-Serving Institutions, community colleges, and think tanks) to engage individuals whose perspectives might otherwise be overlooked. However, although a diverse search committee is a first recommendation, it is not always possible to achieve. Therefore in cases where it is not possible, recruit committee members who are supportive of the equity and inclusion goals, and rotate responsibilities to review selection rubric, process and criteria from the outset. Finally, celebrate and provide credit and visibility to the organizers for efforts.

3. Assess equity goals

Develop an evaluation process to assess whether equity and inclusion goals are met during speaker selection, and share this information in aggregate publicly. This could be done by quantifying and tracking speaker and participant demographics through optional questionnaires. Questionnaires can be designed to measure the chosen equity goals, this could include aspects such as gender, race/ethnicity, career-stage, institution type, sexual orientation, religion. In addition, consider conducting pre-, post and long-term surveys to gather information about experiences of speakers and participants in relation to equity goals, such as perceptions of respect, how much individuals felt valued, and safety. Following review, further recommendations can be made to improve the process for future meetings.

4. Take care when choosing topics

The choice of topic can impact which speakers and audience you attract to a meeting. Prioritize inclusive representation by setting broad topics, and perhaps those topics which have not been historically represented - such as the role of indigenous knowledge in scientific inquiry which could be applicable to a wide number of participants. Avoid tokenism (the practice of making only a symbolic effort, especially by recruiting a small number of people from underrepresented groups in order to give the appearance of inclusiveness), and don't have "diverse" speakers only plan the diversity events or speak on topics related to diversity. Treat speakers as scientists first.

5. Create an equitable schedule

Both the timing and avenues through which speakers can participate has the potential to impact the inclusivity of a conference. Sessions that are scheduled over weekends can pose problems for individuals with dependents. Providing sufficient breaks in schedules for religious observance, medical needs, or for taking care of family needs, e.g., breastfeeding or pumping, can facilitate broader participation. In order to increase access, consider including a mix of in-person and hybrid presentations; the latter can allow participation of speakers who may not otherwise be able to attend due to professional commitments, personal commitments, challenges with travel documents, and/or financial constraints. This increases access for many, such as international speakers, parents of young children, students, postdoctoral researchers, and facilitates participation of individuals from historically under-represented institutions and geographic regions. In addition, when planning concurrent sessions, it is advisable to be mindful of

potential conflicts that might arise, reducing the potential reach of speakers, as participants need to decide between conflicting sessions. Possible conflicts can be minimized for example by knowing well which topics of different sessions are more interdisciplinary and expected to have a wider range of audience.

6. Provide necessary accessibility options to speakers

The conference environment can impact whether speakers decide to attend or not. Efforts should be made to accommodate speakers who use mobility support, have audio/visual impairments, by ensuring the venue has ramps for the stage(s), rooms that are large enough to accommodate devices, and closed captioning, etc. In addition, gender neutral and accessible bathrooms should be made available for speakers to access. Finally, when possible funds should be made available to support recruitment of a diverse set of speakers, covering travel, accommodation, and childcare services when necessary. Establishing the availability and types of support available for speakers prior to opening calls for applications, or sending out invitations, will allow potential speakers to make informed decisions when responding to requests.

7. Identify and invite a diverse set of speakers for plenary or keynote sessions

When inviting speakers, committee members are encouraged to reach outside their networks to identify experts who are underrepresented in a research topic. Recommendations can come from past or present attendees, from those invited speakers that declined the invitation, (specifically for underrepresented groups), from community surveys requesting nominations, by consulting available diversity databases and lists, or from a deep assessment of the literature.

It is advisable to consider aspects beyond publications, such as outreach and education, engagement with policymakers, participation in mentoring programs, and/or advancement of inclusivity efforts in the process.

8. Develop an equitable criteria for selected talks

When soliciting abstracts for sessions, oral presentations and posters, an outreach or promotion plan should be developed to ensure the call reaches communities that are traditionally underrepresented in scientific meetings, such as Indigenous groups, Pacific Islanders, Black or African Americans, Hispanic or Latinos. Promotional material should use inclusive images and language, provide information about the types of support and resources available for attendees, and state that representatives from diverse backgrounds and expertise are encouraged to apply and attend.

Develop a consistent process for evaluation of abstracts, involving transparent selection criteria, a standard rubric, and pre-agreed decision making process. Selection criteria should be well-rounded, clear, and made available to all applicants and to abstract reviewers prior to submission. Speaker selection rubrics from societies and groups (e.g., ASPB, NAASC) to help guide the process.

9. Factor in time for planning

Recognize that an equitable agenda takes time to develop and requires conscientious effort. While inviting a diverse set of speakers is the first step, it must be recognized that women and other underrepresented minorities are more likely to decline invitations for reasons including increased demands on their time, limited funding and support for travel, and family responsibilities. Therefore, organizers are advised to work with speakers to accommodate needs and design conferences around speaker availability when possible, and sufficient time should be allotted to planning a meeting and extending speaker invitations so that alternate speakers can be found if necessary and equity goals still can be met.

10. Be intentional in speaker outreach

If there are speakers known within the community that would be of interest to your attendees, reach out to them directly via a phone call or email. Be sure to include the meeting date, location, size, purpose, and a list of topics of interest. It would also be helpful to include the ideal length of the presentation and the meeting type. Inform potential speakers if financial support or a speaker honorarium is available. Be prepared to address their questions regarding the nature and extent of any support the meeting can provide to encourage their participation. If you are looking to secure more than one speaker for your meeting, you can use the checklist in the Resources section to check the speakers are diverse in terms of gender, ethnicity, and area of expertise.

11. Be respectful of modes of address

To create a welcoming environment check and consistently use speaker pronouns (e.g. she/her/hers, they/them/theirs) and provide options for participants to correct spelling of their names, or to indicate their preferred name, including the placement of diacritical markers (like accents and tildes) that are not necessarily in the English alphabet. In addition, it is advised that session chairs should make efforts to learn to correctly pronounce names and check pronouns ahead of providing introductions during the meeting.

List of Resources

- ASPB - Conference Planning: An instructional guide for scientists. https://aspb.org/meetings/Meeting%20Organizers%20Tool%20Kit%20-%20v1.pdf
- Guide to Organizing Inclusive Scientific Conferences: https://500womenscientists.org/inclusive-scientific-meetings
- SLA - Guidelines and Tips for Promoting Diverse Speakers and Panels https://www.sla.org/wp-content/uploads/2020/07/Guidelines-for-Choosing-Diverse-Speakers-1.pdf
- American Public Gardens Association IDEA Center - Professional Development Manual for Communities: https://www.publicgardens.org/about-us/IDEA
- Ford, H.L., Brick, C., Blaufuss, K. et al. Gender inequity in speaking opportunities at the American Geophysical Union Fall Meeting. Nat Commun 9, 1358 (2018). https://doi.org/10.1038/s41467-018-03809-5
- Sarvenaz Sarabipour (2020) Research Culture: Virtual conferences raise standards for accessibility and interactions *eLife* 9:e62668.
- DiversifyPlantSci: https://rdale1.shinyapps.io/diversifyplantsci/
- Sardelis and Drew. Not "Pulling up the Ladder": Women Who Organize Conference Symposia Provide Greater Opportunities for Women to Speak at Conservation Conferences (2016) PLOS ONE https://doi.org/10.1371/journal.pone.0160015
- Skiles, M., Yang, E., Reshef, O. *et al.* Conference demographics and footprint changed by virtual platforms. *Nat Sustain* 5, 149–156 (2022). Conference demographics and footprint changed by virtual platforms https://www.nature.com/articles/s41893-021-00823-2
- Guide to inclusive language https://www.apa.org/about/apa/equity-diversity-inclusion/language-guidelines
