# Supplementary material for: Making conferences in the plant sciences more inclusive through community recommendations
Source: eLife. 2025 Aug 20;14:e106877. doi: 10.7554/eLife.106877 (PMC12367295; doi:10.7554/eLife.106877)
Supplement: Supplementary file 2. [file elife-106877-supp2.pdf]

April 2023

# Inclusive Conferences Working Group Recommendations

## Executive Summary

Over the last several months the Inclusive Conferences Working Group (ICWG), composed of twenty-three members representing all seven RCN plant science societies and five facilitators from the RCN Steering Committee, developed a document which details an extensive set of recommendations that support inclusive and accessible conference environments. In December 2022, the Inclusive Conferences Working Group sent these recommendations to each organization for their comments and feedback.

In March 2023, the Inclusive Conferences Facilitators reviewed and integrated this feedback into a 10-page "Executive Summary". For reference, the consolidated feedback from all organizations can be found here. This Executive Summary was reviewed by the Inclusive Conference Working Group and Movement Consulting.

This Executive Summary is a **high-level overview** of the recommendations provided by the Working Group. This summary can serve as a guide when planning your organization's annual meetings and other conferences. For the full recommendations, please see this document.

A few things to note:

- ROOT & SHOOT has scheduled a Bystander Training for members of the RCN, provided by the ADVANCEGeo Partnership. These workshops will be held on Tuesday, June 27 from 11am-2pm EDT and Monday, July 10 from 3-6pm EDT. Steering Committee liaisons will be in charge of coordinating sign-ups from their organizations.
- In the coming months, ROOT & SHOOT will be exploring the possibility of a joint ombudsperson for our conferences.
- There was general consensus that the Community Agreement should be adopted by each organization in addition to, or in place of, their existing Code of Conduct.
- If desired, arrangements can be made for a few members of the ICWG to be available to society leadership, to answer questions or further describe the process.

# Community Agreement

## I. Goal of the community agreement

The "Plant Sciences Community", as defined in this document, is a network of professional membership organizations that represents those with a focus on advancing a wide range of plant-oriented science. The goal of this community agreement is to present a collective vision for how we, the Plant Sciences community, want to define our values and set expectations and measures for accountability for ourselves, our staff, and all affiliated participants of our collective gatherings.

## II. Community Values

[CONFERENCE NAME] strives to ensure the full participation, safety, and support of all participants. We work to uphold inclusive and welcoming practices that oppose racism and promote racial equality and decolonization[1]; to support queer, trans, non-binary, and gender expansive scientists; to provide equitable access for scientists with disabilities; to invite and uplift contributions by all scientists, irrespective of religion, belief systems, and cultural backgrounds; to elevate the work of researchers in marginalized communities and institutions; to strive for equitable access to resources in those communities; and to remove barriers for those whom science has historically excluded.

## III. Conference Participant Agreement

As a participant in [CONFERENCE NAME], I agree to do my part in building a welcoming and inclusive conference environment by:

- Holding myself and others accountable to the community values while at the conference, whether attending formal events or unofficial activities.
- Critically evaluating my own implicit biases and reflecting on how they impact my interactions with others.
- Treating others with civility and actively working to avoid, prevent, and stop harassment, discrimination, and behavior that is exclusive, threatening, intolerant, abusive, or violent.
- Upholding the highest standards of scientific and academic integrity during the conference, recognizing past and present contributors to science, while acknowledging that science, as practiced by humans, can be biased and has harmed marginalized communities.
- Evaluating the work of colleagues equitably—with open-minded fairness and respect, and being aware that power dynamics can introduce bias.
- Encouraging and promoting climates where multiple scientific perspectives may be freely expressed and valued.

This agreement was developed by a group of plant scientists as part of the NSF-funded Root & Shoot Research Coordination Network (NSF RCN LEAPS #2134321). By attending [CONFERENCE NAME], you agree to abide and uphold this living community agreement in its current form while contributing to amendments in the future.

### Appendices and links to reporting documents

A: Justification for community agreement

[1] Decolonization refers to the active process of examining, deconstructing, and dismantling earned privilege resulting from historical injustices.

B: Desired Outcomes and Unacceptable Behaviors
C: Additional References

## Reporting Structure

*Please note this is a summary of the Reporting Structure section; the full recommendation can be found [here](#).*

To promote inclusiveness at conferences it is important that both conference organizers and attendees be aware of the many dimensions of equity and inclusiveness and that there be systems in place to ensure that both organizers and attendees are held accountable for pursuing policies and behaviors that promote both. As part of this process, the working group recommends that ROOT & SHOOT develop a Pre-Conference survey for all conferences organized by the seven participating plant societies that would serve as a one-stop shop for potential attendees to assess conferences on multiple dimensions of equity and inclusiveness (e.g., diversity of speakers, location, accommodations for those with disabilities, low-cost lodging options, registration fees, etc.). Results from such a survey would incentivize conference organizers to address issues of equity and inclusiveness while helping attendees select conferences that best fit their needs. It would also enable societies to advertise the multiple steps they have taken to promote equity and inclusiveness.

Once attendees are at a conference, it is expected that all in attendance will abide by the Community Agreement. To ensure accountability and compliance with the Community Agreement, organizers must put in place a clear reporting structure that is easy for attendees to navigate and which results in appropriate actions being taken. To accomplish this goal, the ICWG recommends the following actions:

- Misconduct/community agreement violations should be explicitly defined, and the information should be made readily available on the website of every plant biology society that is part of the ROOT & SHOOT RCN, as well as on the conference website and within the meeting app (if used). Additionally, consequences for violating the community agreement should be laid out.
- Each society should have a mechanism for the implementation and oversight of reports of interpersonal conflict and violence (ICV; an umbrella term for acts such as sexual assault, physical assault, and sexual misconduct, as well as non-physical acts such as the use of slurs, racist, ableist, sexist, homophobic, transphobic, anti-Black, anti-Indigenous, Islamophobic, or anti-semitic language, inappropriate or harmful gestures, and coercive actions (e.g., using one's status to make another person do something against their will)).
- The seven societies should adopt a standardized community agreement violation reporting form[2]. These reports should feed into a centralized data server for all seven R&S societies. This form should be explicit about who will have access to the information in the report, whether certain information must be reported to legal authorities, and who will have access to the name of the reporter.
- Each conference should appoint an independent (not a society member) point person to function as an ombuds-person or mediator for when conflicts arise. Ideally, R&S could contract with someone to be on call to all seven societies during their conferences for when such interventions are needed (presumably from a remote location in this model). Such a shared ombuds-person could then be responsible for monitoring submissions of Community Agreement violation reports and following up with anyone filing a report. In situations in which laws may have been broken, the ombuds-person/mediator would assist the victim with reporting the crime to relevant authorities.
- Provide both confidential and non-confidential options for reporting, ideally through an easy to locate electronic means (e.g., through a link on conference website or meeting app),

paper means (e.g., a reporting box), phone number (e.g., a HELP line) and in-person means. In this context, ‘confidential’ means that the identity of the reporter would not be shared with society members but would be known to the independent ombuds-person/mediator so that follow-up can occur.

- ROOT & SHOOT should provide training to the officers and staff of the seven R&S societies in a Bystander Intervention program that encourages individuals witnessing violations of the Community Agreement to intervene when they feel it necessary.
- Each society should draft and implement a plan for directly addressing and resolving reports of ICV during the conference, prioritizing the needs of the reporter, including referral to local and regional support centers if appropriate (e.g., in the case of sexual assault).
- Consequences: It is important to highlight the consequences of community agreement violations. Consequences should be developed for each case after sincere discussion between the accused, the reporter, and the mediator. Potential consequences could include a) a warning, b) required training commensurate with the violation, c) removal from the meeting without a refund, d) prohibition from attending future meetings of any of the seven R&S societies, e) removal from society membership, f) reporting to their employer, and g) reporting to law enforcement when appropriate.
- Create an ICV-specific post-conference reporting mechanism. An invitation to report incidents should be sent out immediately following the conference, separate from any other ‘satisfaction’ surveys. The process of reporting and follow up should be transparent in the introduction to this invitation so that reporters know their identities will be kept confidential, how the information provided will be used, and that they will receive support. It will be important, though, for societies to make public the number and types of incidents reported so that progress can be tracked from year to year. This system should be standardized and shared across the seven R&S societies.

    - Post-conference ICV reports (with reporter identifications removed) should be reviewed by a board or committee that encompasses society leadership, staff, and general members. If funding is available, this team of reviewers should be composed of ROOT & SHOOT volunteers from other societies. This would assure a degree of anonymity as well as reduce possible bias from people who participate within the society group. Reports would be collected and reviewed, and situations that require immediate assistance such as issues of sexual violence, or racism and discrimination can be prioritized, bringing in the help of legal counsel if laws have possibly been broken.
    - Committee members should work with the ombuds-person or an external program to prepare a plan for addressing the incident. Should this require legal involvement, the ombuds-person would be responsible for reaching out to the reporter to determine if they would like to press formal charges and help them do so with the local authorities.
    - If incidents do not require legal action, a plan for addressing the incident will be drafted and shared with the reporter and the accused, with opportunities for each to respond. The committee will work with each to reach a satisfactory resolution. Resolutions could include consequences such as those listed in the "During Conference" section of this document.

# Transparent Site Selection

*Please note this is a summary of the Transparent Site Selection section; the full recommendation can be found here.*

Transparency in conference site selection is essential for society members to understand the range of elements impacting equity and inclusion when a choice of site is made. In addition, there are other practical aspects of organizing a successful conference that must be considered: for example, venue size, cost of accommodations and level of local support by society members. However, supporting participation by a diverse society membership is crucial, and the range of member experiences must be considered when choosing conference location. Dissemination of specific criteria used to select a conference site should be conveyed to society membership on an annual basis. Given that sites are selected several years out, this also helps communicate rationale for choices made, even though changes in society personnel and site environment may change over that period of time.

This summary provides an abbreviated checklist of topics and questions that are recommended for ROOT & SHOOT RCN societies to use to consider important criteria in site selection. This cross-section of factors should be available to members (e.g., on the society website), used to guide the selection process in site assessment, and eventually, be provided at the conference (e.g., via a conference app or other mechanism). The goal is to provide a framework for evidence-based decisions for each site choice, and at the time of the conference, use the same framework to inform society members transparently regarding specific aspects of a chosen site, assisting in a decision whether to attend. Annual surveys to determine the response of membership to each conference site, particularly to the factors listed in this framework, are also recommended. Due to the breadth of factors listed, selection decisions will likely weigh positive and less-positive aspects - a long-term view that balances all factors over several years may be necessary.

**Overarching Considerations**

- Who were the members of the site selection committee, and is the process for determining committee membership transparent? How is the final decision made (e.g., who researches, who recommends, who votes?)
- Were multiple sites considered? Across the years, is the conference held in different geographic regions to increase access and ensure a diverse range of surrounding populations?
- Is survey information regarding society membership needs and preferences collected on a regular basis, to most effectively inform site selection and conference planning?

**Location and Setting**

- Have local or state governments enacted laws that may endanger attendees based on their identity?
- Will the local community derive benefits from the conference (e.g., invite community members to attend, society-sponsored outreach efforts)?

- Are there local members of the community that can help build a list of recommendations that attendees can use to make decisions about food, lodging, etc. For example, Black-owned restaurants/catering, local/small businesses.
- Are there sufficient food options nearby that can accommodate a variety of dietary needs?
- Are there affordable, nearby lodging options that can accommodate diverse needs (e.g., single rooms and shared rooms)?

**Transportation**

- Is there an international airport in the area, accessible by budget-friendly airlines?
- Are there affordable options other than air travel for reaching the site (e.g., train, buses)?
- Is there public transport between the conference site and airport? And for moving around the city? Can the conference help support attendee use?
- Does the conference or venue provide mechanisms for attendees to easily and safely return to accommodations if events go late into the evening?

**Venue**

- Does the venue comply with Americans with Disabilities Act (ADA) accessibility standards (or analogous international standards)? What disability provisions are there in meeting rooms and poster session space (e.g. wheelchair accessibility)?
- What child care accommodations are available at the venue? Accommodations for other needs (e.g., quiet rooms, lactation rooms)?
- Does the venue have enough space for non-overlapping social events (e.g., affinity group gatherings)?
- Does the venue adhere to fair hiring practices and equitable treatment of staff (e.g., is there any history of worker disputes)?

**Sustenance**

- Did food options at and near the venue reflect the diet diversity of attendees (vegan, vegetarian, gluten-free, etc)?
- What is per diem for the host city, and do local food establishments have options that fit within the per diem budget?
- Are food vendors available at all reasonable hours? Are there any seasonal changes in business hours that might affect conference attendees?

# Conference Accessibility

*Please note this is a summary of the Conference Accessibility section; the full recommendation includes a more robust check-list of items to consider, and can be found here.*

Accessibility is the foundation of an equitable and inclusive conference. Indeed, inclusive societies have an obligation to use their influence to continually push for increased accessibility. Accessibility should be part of the conference planning from the start: site selection choice, budget development, and program structure. Here, conference accessibility is divided into five “environments”: Physical, Audio/Visual, Family, Economic, and Wellness. It should be noted that certain accessibility options (e.g., economic vs physical environment) may be in direct conflict, so a long-term view that balances choices over several years may be necessary.

***Economic Environment***. The gap between the well-resourced and resource-poor is exacerbated unless inclusive conference approaches are employed. Participating in scientific conferences is an expected part of being a scientist and considered vital especially to those earlier in their career, including students, postdoctoral scholars, and new faculty. Selecting conference locations that provide lower cost options for travel and accommodations, as well as conference registration fee structures that enable subsidizing of early career researchers and those from under-resourced countries can enable broader participation. Communicating your priorities early to your community will convey your society’s inclusive values.

***Physical Environment.*** The physical environment of the conference includes the conference venue (where presentations and the scientific and social events will take place) as well as the conference hotel or other overnight lodgings; attendees will need to travel between and navigate these spaces during the conference, so it is imperative that organizers assess the continuum of these physical spaces to ensure accessibility.

***Audio/Visual Environment.*** Worldwide, 8.5% of people have some form of hearing loss and/or vision impairment. In the United States, 30% of working professionals have an audio/visual disability (20% are deaf or hard of hearing and 10% have some degree of vision loss). This is a considerable number of people who require some type of accommodation in order to benefit fully from conference participation.

***Family Environment***. Caregivers are a major part of the workforce, and conferences get higher attendance rates when they offer accommodations to bring dependents along, or support caregivers to take care of their dependents at home.  We point to conference characteristics that would help ensure that the conference is family-friendly and inclusive to caregivers.

***Wellness Environment.*** Wellness can be an all-encompassing term used to describe one’s physical, mental, and emotional wellbeing. In the context of developing inclusive conferences, preparations can be made to promote the safety and comfort of all participants, including those who are often excluded from conferences because they require particular arrangements and may not otherwise have the physical and/or financial ability to attend the conference.

**Action Items for Inclusive Conferences:**

***Immediate***

- Include several questions during the registration process to determine what types of accommodations are needed and give input with their accessibility needs or concerns.

- Conference websites should include an “accessibility” section, visible on the main menu so users have just one click to locate it, that makes clear the accessibility features (map, info/photos of meeting rooms) that will be provided and invites community contact to resolve potential questions and issues. When the recommendations in this document cannot be met, clearly indicate relevant information on the accessibility page so that participants can plan accordingly.
- Site visits ahead of the conference to ensure appropriate mobility options for all mobility types (i.e., ramps, adequate signage, wide aisles at poster sessions).
- Quiet or low stimulation rooms on site.
- Ample break time between sessions, as the conference environment can be especially overwhelming and overstimulating to neurodivergent people.
- Accessibility seating in all rooms along the aisles and clearly labeled.
- Several microphones should be available in every conference room.
- Offer an online workshop several weeks to a month before the conference for presenters to provide guidelines on how to make their presentation accessible. Alternatively, or in parallel, develop an RCN-wide shared video covering this topic, and make it available on all conference websites.
- Ensure that a lactation room or portable lactation room is available at the venue.
- Allow presenters with attending dependents to schedule their talks at times that would better accommodate their family obligations. You can survey invited speakers in advance to help structure the program.
- Offer non-alcoholic beverages for people who do not consume alcohol. Consider policies that address when alcohol is made available during the conference.

***Long-term***

- Live caption writer on site and/or remotely.
- Starting as early as the initial budgeting and site selection process, each step should consider how options may affect broad participation. For example, you may forgo offering ‘more-resourced’ invited speakers complimentary registration and reserve additional budget for support of less-resourced invited speakers and other similar participants.
- Offer low- to mid-priced conference lodging (e.g., university dormitories for summer conferences) and travel options.
- Volunteers to help at the conference in exchange for funding support.
- Provide information on affordable options for meals not included in the conference fee.
- The conference website should follow the W3C Recommendation from the Web Content Accessibility Guidelines (WCAG) 2.0 and be tested for functionality before sharing.
- Consider providing sign language services on-site (and include good lighting so the audience can see the person signing) or remotely.
- CART captioning (in the room and online).
- For international conferences, we recommend providing access to the conference webpage and documentation on a variety of platforms, taking into account some countries' webpage restrictions.
- Provide an assistant to help advance slides for presenters who may have visual impairments; provide an assistant who can read posters for blind/low vision attendees and/or provide tactile maps/audio options for posters.
- Offering a virtual meeting component (or recorded talks) to make talks more accessible
- Offering options to attendees with dependents is essential (e.g., offering childcare on-site, providing grants to attendees to cover expenses associated with bringing a caregiver or paying for additional caregiving costs at home).

- Establishing protocols for social events/interactions with regard to COVID and other health concerns, and preferences for interaction, will encourage participation from a wider community and will benefit the community overall.
- Offer hybrid options - offering a virtual component will enable access to the meeting for my groups such as people with families, disabilities, financial need, visa and travel concerns. If providing a hybrid option, assign a moderator for talks to monitor and repeat questions, and provide clarifications. Encourage closed-captioning for all recorded and live talks. Host virtual social events for off-site participants.

## Speaker Selection & Equitable Programming

*Please note this is a summary of the Speaker Selection and Equitable Programming section; the full recommendation can be found here.*

- Organizations should **survey their community** (speakers/presenters and prospective participants) during the conference development phase to gain input on their priorities to help guide decisions.
- **Funding** - develop a budget that prioritizes early career scholars, participants from less-resourced institutions, and/or attendees from less wealthy nations.

    a. Individuals with the least resources should be prioritized for discounts, or for direct funding, to enable their participation (transparency in the communication and clearly conveying discounted options for invited speakers)
    b. Offer invited speakers a standard benefit, and give them the option to opt-in to a previously-extended benefit. In other words, the invitation could extend free registration (a standard benefit), but make an additional benefit (e.g., travel subsidy) by opt-in request (confidentiality should be a priority).

1. **Make a commitment to assemble as diverse a group of speakers** as possible in terms of but not limited to race, gender identity, career stage, sexual orientation, age, disability, ethnicity, immigration status, national origin, geographic area, religion, socio-economic status, and institute type[2].
2. **Form an organizing committee that is diverse to invite a diverse set of speakers**. Consider reaching out to different kinds of institutions (e.g. Minority-Serving Institutions, community colleges, and think tanks) to engage individuals whose perspectives might otherwise be overlooked. Add more career-stage diversity in the organizing committee (not only among the speakers).
3. **Assess equity goals during speaker selection, and share this information in aggregate publicly**. This could be done by quantifying and tracking speaker and participant demographics through optional questionnaires as well as pre-, post and long-term surveys to gather information about the experiences of speakers and participants in relation to equity goals. Following review, further recommendations can be made to improve the process for future meetings.
4. **Be intentional when choosing topics because they can impact which speakers and audience you attract to a meeting**. Prioritize inclusive representation by setting broad topics, and perhaps also topics that have not been historically represented - e.g. the role of Indigenous knowledge in scientific inquiry - and could be applicable to a wide number of participants. Consider implementing mechanisms to gather community input on topics to be included. Avoid tokenism – don't have speakers of marginalized identities only plan the diversity events or speak on topics related to diversity. **Treat all speakers as scientists first.**
5. **Create an equitable schedule,** as both the timing and avenues through which speakers can participate have the potential to impact the equity and inclusivity of a conference.
6. **Provide necessary accessibility options to speakers** - mobility support, audio/visual impairments, gender-neutral and accessible bathrooms. If funds are available, provide caretaker and/or childcare services when necessary.

[2] A challenge for the future will be how to collect and store this data anonymously.

7. **Identify and invite a diverse set of speakers for plenary or keynote sessions.** It is advisable to consider aspects beyond publications, such as outreach and education, engagement with policymakers, participation in mentoring programs, and/or advancement of equity and inclusivity efforts in the process. Deprioritize speakers who have recently presented at the same or related meeting.
8. **Develop equitable criteria for selected talks and abstracts.** When soliciting abstracts for sessions, oral presentations and posters, an outreach or promotion plan should be developed to ensure the call reaches communities that are traditionally marginalized in scientific meetings such as Black or African Americans, Hispanic or Latine, Indigenous groups, Pacific Islanders, people with disabilities, women, and underrepresented groups in the respective country of meeting origin. In addition, develop a consistent process for the evaluation of abstracts, involving transparent selection criteria, a standard rubric, and a pre-agreed decision-making process. Speaker selection rubrics from societies and groups (e.g., ASPB, NAASC) should be relied upon to help guide the process.
9. **Factor in time for planning** and recognize that an equitable agenda takes time to develop and requires conscientious effort. While assembling a diverse set of speakers is the first step, it must be recognized that marginalized people are more likely to decline invitations for reasons including increased demands on their time, limited funding and support for travel, and family responsibilities.
10. **Be intentional in speaker outreach**.If there are speakers known within the community that would be of interest to your attendees, reach out to them directly via a phone call or email. Inform potential speakers if financial support or a speaker honorarium is available. Be prepared to address their questions regarding the nature and extent of any support the meeting can provide to encourage their participation.
11. **Be respectful of modes of address** - use speaker pronouns (e.g., she/her/hers, they/them/theirs) and provide options for participants to use the title (Dr.), correct the spelling and pronunciation of their names, or to indicate their preferred name.

# Appendix

## Appendix 1

**Suggested items to include on a conference scorecard:**

- Does the society have a community agreement (or equivalent) that describes how they resolve infractions?
- Has the society committed to balancing speakers based on: career stage, gender, and geography?
- Will the meeting offer support for those with additional audio or visual needs? (e.g., ASL interpreter, closed captioning, slides available)
- Does the meeting offer support for parents or those with caregiving obligations? (e.g., lactation room, family activities, daycare, subsidies to pay additional costs for caregiving at home while attending meeting)
- Does the meeting offer a dedicated quiet space for use by neurodivergent attendees, people with auditory processing disorders, families, etc.
- Are hybrid options available? If so, are they interactive (beyond simply passive viewing of recordings)
- Is the city/area relatively safe for our most vulnerable members – nowhere is 100% safe for anyone, but there could be some metrics/ratings available (e.g., statistics on hate crimes and police-mediated violence)
- Does the host country/state have laws that target members of LGBTQ+ communities and/or identify homosexuality as illegal?
- Is the area around the meeting site safe for walkers in terms of side-walks, pedestrian crossings, and crime?
- Does the host city have a transit system with wheelchair accessible options?
- Are lower cost lodging options available for this conference?
- Are registration fees for early career researchers discounted?
- Are 'scholarships' for those attending from under-resourced countries available?
- Can early career researchers apply for travel awards, and if so, when is the deadline for applications?
- Has society delivered on past promises? This would be blank in year one, but this is where the post-meeting surveys could be integrated. (e.g., addressing reports of microaggressions and blatant hate speech)

**Standardized community agreement reporting form:**

- It would be helpful for the societies involved with Root and Shoot to develop a unified form for reporting macro and micro-aggressions. We include a draft of what this may look like but invite the leadership to consult with experts and improve upon this. We feel it is particularly important to include some version of the question, "what would you like done", as there are times that people will want follow-up action and times where just bringing the incident to the attention of the conference or ROOT & SHOOT is enough. This form should be explicit about who will have access to the information in the report, whether certain information must be reported to legal authorities, and who will have access to the name of the reporter.

1. Are you a participant at CONFERENCE NAME?

2. Is the person for whom this report is about a participant at the CONFERENCE NAME?
3. Date of reported incident:
4. Location of reported incident (e.g. within the convention center, hotel, or off site):
5. Description of the incident and context (please provide as many details as possible):
6. Were you directly involved in the incident or indirectly involved (observed or heard about via second hand knowledge)?
7. Offending individual's name (or description if name is not known or you wish not to disclose):
8. Did you or anyone else ask the offending individual to stop or address their behavior directly?
9. Desired outcome or resolution:
10. What is your name and contact information so that we can follow up with you regarding this incident?
    a. Name:
    b. Email:
    c. Phone number:

# Inclusive Conferences Working Group Members

| | | | |
|---|---|---|---|
| Group A - RCN-Wide Community Agreement | Burcu Alptekin | University of Wisconsin-Madison | American Society of Plant Biologists (ASPB) |
| | Carrie Tribble | University of Hawai`i at Mānoa | American Society of Plant Taxonomists (ASPT), Botanical Society of America (BSA) |
| | Gaurav Kandlikar | University of Missouri Columbia | Botanical Society of America (BSA) |
| | Hank Bass | Florida State University | Maize Genetics Cooperation (MGC), EU INDEPTH (expired, but ongoing) COST Action group |
| | Corri Hamilton | University of British Columbia | American Phytopathological Society (APS), American Society of Plant Biologists (ASPB) |
| | Marcia Puig-Lluch | | ROOT & SHOOT Steering Committee Representative (ROOT & SHOOT) |
| Group B - Reporting Structure | R. Shawn Abrahams | Yale University | American Society of Plant Taxonomists (ASPT), Botanical Society of America (BSA) |
| | Kanwardeep S Rawale | Geneshifters | American Society of Plant Biologists (ASPB) |
| | Andrew Read | University of Minnesota | International Society for Molecular Plant Microbe Interactions (IS-MPMI) |
| | Theo (Chelsea) Newbold | The Pennsylvania State University | American Phytopathological Society (APS) |
| | Morgan Gostel | Botanical Research Institute of Texas | American Society of Plant Taxonomists (ASPT), Botanical Society of America (BSA) |
| | Roger Innes | | ROOT & SHOOT Steering Committee Representative (IS-MPMI) |
| Group C - Transparent Site Selection | Cody Coyotee Howard | Oklahoma State University | Botanical Society of America (BSA) |
| | Michael Moody | University of Texas at El Paso | American Society of Plant Taxonomists (ASPT), Botanical Society of America (BSA) |
| | Annarita Marrano | AgBioData/Phoenix Bioinformatics | American Society of Plant Biologists (ASPB) |
| | Kasia Dinkeloo | Univeristy of Texas at Austin | American Society of Plant Biologists (ASPB) |
| | Mindy Findlater | University of California, Merced | American Society of Plant Biologists (ASPB) |
| | John Fowler | | ROOT & SHOOT Steering Committee Representative (MGC) |
| Group D - Conference Accessibility | Patricia Baldrich | Donald Danforth Plant Science Center | International Society for Molecular Plant Microbe Interactions (IS-MPMI) |
| | Adriana Hernandez | Cal Academy of Sciences | American Society of Plant Taxonomists (ASPT), Botanical Society of America (BSA) |
| | Imeña Valdes | Northwestern University/Chicago Botanic Garden | Botanical Society of America (BSA) |
| | Joanna Friesner | The Danforth Plant Science Center & NAASC | North American Arabidopsis Steering Committee (NAASC) |
| | Melanie Link-Perez | University of Tampa | Botanical Society of America (BSA) |
| | Leslie Holland | | ROOT & SHOOT Steering Committee Representative (APS) |
| Group E - Speaker Selection & Equitable Programming | Steven Burgess | University of Illinois at Urbana Champaign | American Society of Plant Biologists (ASPB) |
| | Eric Wada | Folsom Lake College | Botanical Society of America (BSA) |
| | Sakina Elshibli | University of Helsinki | American Society of Plant Biologists (ASPB) |
| | Denita Hadziabdic-Guerry | | ROOT & SHOOT Steering Committee Representative (APS) |
