# Supplementary material for: Making conferences in the plant sciences more inclusive through community recommendations
Source: eLife. 2025 Aug 20;14:e106877. doi: 10.7554/eLife.106877 (PMC12367295; doi:10.7554/eLife.106877)
Supplement: Supplementary file 3. [file elife-106877-supp3.pdf]

This document represents a subset of the recommendations of the ROOT & SHOOT Inclusive Conferences Working Group. We encourage you to read the full report.

**Site Selection Recommendations**

**Overview**

Conference site selection decisions have traditionally focused issues such as venue price and size and the level of local support by society members to host the conference. However, the needs and priorities of *all* conference attendees must be considered when choosing the conference location. **Transparency** in conference site selection is essential for society members to understand how equity and inclusion has been considered when evaluating conference sites.

Here we provide a set of questions that the organizing committee should consider as they evaluate sites. We recommend that the site-specific information relevant to these queries be shared on the conference website and in the conference App, supplemented with links to local information, to make it easy for society members to decide for themselves how safe, inclusive, welcoming and accessible the site will be for them.

**Overarching**

- *Who were the members of the site selection committee and how were they selected?*
- *Were multiple sites considered? Or just one?*

**Location Setting**

- *Does the surrounding population reflect the diversity of conference attendees?*
- *Have local or state governments enacted laws that may endanger attendees based on their identity?*
- *Are reproductive services available including emergency abortion care?*
- *Are people who know the area available to provide on-the-ground advice and recommendations?*
- *Are there sufficient food options nearby that can accommodate a variety of dietary needs?*
- *Are there affordable, nearby lodging options that can accommodate diverse lodging needs (e.g., affordable single rooms as well as shared rooms)?*
- *Have you provided mechanisms for attendees to easily and safely return to accommodations if events extend late into the evening?*

**Venue (building or building complex where event will be held)**

- *Are there any known ownership history or current practices that have led to worker disputes in relation to equitable treatment of staff? Does the venue adhere to fair hiring practices?*
- *Does the size of the venue not only consider space for professional presentations and meetings to accommodate the society membership but also provide*

*space/rooms that can be utilized for special needs, such as quiet rooms and lactation rooms?*

- *What criteria were used for selection of volunteers (was diversity considered) at the venue and how will they be compensated for their time (meals, registration, free time)? Will accommodations be made for their attendance at talks, etc.?*
- *Does the venue comply with Americans with Disabilities Act (ADA) accessibility standards and is it able to provide additional accommodations if needed? What provisions are there in conference and meeting rooms and poster session space (e.g. wheelchair accessibility)? Are signs and websites accessible?*
- *What day care accommodations are available at the venue?*
- *Have you built in enough time, and does the venue have enough space for non-overlapping social events?*
- *Have you contacted local community members and invited them to participate so they can both benefit from and contribute to the event?*

**Sustenance**

- *Did organizers evaluate food options within and surrounding the conference site and lodging?*
- *Do options reflect the diet diversity of attendees (vegan, vegetarian, Halal, Kosher, gluten-free, etc.)?*
- *What is per diem for the host city, and do local food establishments have options that fit within the per diem budget?*
- *Are food vendors available at all reasonable hours?*
- *Are there any seasonal changes in business hours that might affect conference attendees?*

**Transportation**

- *Is there an international airport in the area accessible by budget-friendly airlines?*
- *Are there other affordable options than air transportation for reaching the site (e.g., train, buses)?*
- *Is there public transport to travel to/from the conference site and around the city? If yes, are you providing free daily passes for public transport? Which are the criteria for obtaining these free passes?*
