# Supplementary material for: Making conferences in the plant sciences more inclusive through community recommendations
Source: eLife. 2025 Aug 20;14:e106877. doi: 10.7554/eLife.106877 (PMC12367295; doi:10.7554/eLife.106877)
Supplement: Supplementary file 4. [file elife-106877-supp4.pdf]

INTRODUCTION

Accessibility is the foundation of an inclusive conference. In addition to its social justice aspect, accessibility is a fundamental requirement; for example, 1 in 4 people in the United States have a disability according to the CDC (https://www.cdc.gov/ncbddd/disabilityandhealth/infographic-disability-impacts-all.html). Ideally, professional societies will choose conference locations that will already have in place many of the suggestions below; however, the reality is that some sites are unable to provide full accessibility options. In time, conference sites may increase their accessibility options as conference organizers request and demand accessibility features. **Indeed, inclusive societies have an obligation to use their influence to continually push for increased accessibility. Accessibility should be part of the conference planning from the start: site selection choice, budget development, and program structure. Inclusive societies have accessibility as a core value.**

The best accessibility plans will fail to achieve their objectives if they aren't clearly communicated, in advance, to communities. **What is outlined in this document is offered as 'basic best practice' accessibility features, regardless of whether they are requested or not. Organizations should survey their community (speakers/presenters and prospective participants) during the conference development phase to gain input on their priorities to help guide decisions. Then, include several questions during the registration process to determine what types of accommodations are needed.** These questions will enable the organizers to provide additional accommodations as needed.
Conferences should establish communication mechanisms (e.g., email address, Google form, Slack, etc., that are monitored and responsive) and invite the community to give input as early as possible with their accessibility needs or concerns. Make clear that you are committed to enabling an inclusive environment and broad participation, and then follow-through on that commitment.

This section is divided into five accessibility "environments": Physical, Audio/Visual, Family, Economic, and Wellness. It includes recommendations and check-lists to enable broad and inclusive planning. Conference websites should include an "accessibility" section, visible on the main menu so users have just one click to locate it, that makes clear the accessibility features you will provide and invites community contact to resolve potential questions and issues. When the recommendations in this document cannot be met, clearly indicate relevant

information on the accessibility page so that participants can plan accordingly. Some participants may need to make individual arrangements, some of which should be financially supported by the conference through an “accessibility” or “equity” fund. While dedicated fundraising for accessibility is recommended, a complementary approach is to designate a percent of each registration fee for the “equity fund”. It isn’t always straightforward to determine the level of equity fundraising a society should pursue; some things are easier to quantify (e.g., 25 awards of $500 each for early career participants), whereas the accessibility needs of individuals may vary. Surveying for community input, prior to and during conference development, may help guide appropriate budget planning. Ultimately, decisions may need to be made based on limited resources and societies that engage their members in this discussion will better serve their community. Ideally, such discussions will raise awareness, and, by tapping into collective community creativity, develop new solutions.

(1) ECONOMIC ENVIRONMENT

Participating in scientific conferences is an expected part of being an academic scientist and considered vital especially to those earlier in their career, including students, postdoctoral scholars, and new faculty. There are other groups that also particularly benefit from the networking and collaborative opportunities at conferences, such as faculty at primarily undergraduate institutions (PUI) and members of groups that are under-represented in STEM. Paradoxically, these groups tend to be those with the fewest resources; thus, the gap between the well-resourced and resource-poor is exacerbated unless inclusive conference approaches are employed. Early in the conference planning process, societies should develop approaches and options to enable broader participation, with a significant priority given to those with the greatest financial need. Starting as early as the initial budgeting and site selection process, each step should consider how options may affect broad participation. This is a “triage” opportunity; in other words, consider how the budget affects those with the most, or the least, financial need.

A common practice has been to extend financial support to invited speakers who tend to be mid- or late-career, well-established, and often tenured, faculty; however, they tend to be the most-resourced community members. It is recommended to develop a budget that prioritizes early career scholars, participants from less-resourced institutions, and/or attendees from less wealthy nations. For example, you may offer ‘more-resourced’ invited speakers complimentary registration and reserve additional budget for support of less-

resourced invited speakers and other similar participants. The conference can be structured using various approaches so that those with the least resources are prioritized for discounts, or for direct funding, to enable their participation. Communicating your priorities early to your community will convey your society's inclusive values and, if the revised budget is a significant shift to your standard operations, may clear up confusion. For example, if your society revises the budget such that invited speakers will be offered reduced benefits from previous norms, conveying this change, and the rationale behind it, provides transparency. The new budget approach should be conveyed explicitly in speaker invitations. One possible approach is to offer invited speakers a standard benefit, and give them the option to opt-in to a previously-extended benefit. In other words, the invitation could extend free registration (a standard benefit), but make an additional benefit (e.g., travel subsidy) by opt-in request. It should be made clear that their choice will remain confidential, and if the speaker is in need of the travel subsidy, that they can easily opt-in to receive it by stating "I request the travel subsidy" when they reply to accept the invitation.
These conversations would remain private between the society and the invited speaker, and thus, speakers shouldn't feel undue pressure to decline funds they truly need.

1. Registration fees should be tiered and subsidized for less-resourced participants
   a. **Discounts or subsidies for participants from developing countries**, if an international conference. Investigate the relative funding rates of students and faculty in countries or regions that may want to attend. Consider offering discounted rates to attendees from such regions (e.g., the Global South). Raise unrestricted funds to support such attendees (i.e., defrayed or free, registration).
   b. **Set fees according to career level**, charging early-career participants less than later-career participants. Those with more resources subsidize some of the cost of lower-resourced individuals (e.g., students rates vs higher faculty & industry rates).
      Keep in mind additional categories that fall between the lower student & postdoc tiers and higher tenure-track faculty tier, such as research scientists, non-profit staff, and teachers/faculty from Primarily Undergraduate Institutions (PUIs).
   c. **Consider the cost/benefit of everything that goes into the registration fee cost**. If the conference includes activities, charging the cost evenly in registration fees may make the fee too expensive for many. If the activity is ticketed separately, the charge is borne by those with resources; however, this generates a divide between the well-

resourced and the less-resource attendees. Some approaches: tiered level of activity fee; fundraising to subsidize activity costs, combined with tiered pricing; fundraising for attendees that request a fee waiver or reduced ticket price; and develop less expensive activities, to be more inclusive. These approaches apply to all costs, e.g., management services, catering, and facilities.

   d. **Offer a reduced (or waived) rate for a caregiver for a disabled participant who needs to bring a companion/aid with them.** The fee could be waived entirely, if possible, or could be limited to some core costs such as meals, coffee breaks, or staff management fees.
   e. **Consider adding a partial-meeting fee for participants that can only attend several days.** Some participants are only able to attend a conference for a day or two due to family obligations, personal disability or illness, or other responsibilities. While the success of conferences, including financially, relies on full participation by most people, allowing a subset of participants to opt-in to a single or multiple day fee could expand participation.

2. Conference participation awards should prioritize those with fewer resources and support the objective to diversify participation.
   a. **Offer grants for underrepresented communities with financial need**, e.g., members of underrepresented groups by race or ethnicity, gender, sexual orientation, gender identity, historically under-served and under-resourced institutions (e.g., Primarily Undergraduate Institutions, public regional universities, or HBCUs), first generation to attend college, etc., collectively “minoritized” participants. Members of underrepresented groups or under-resourced institutions are less likely to have the resources to participate fully in conferences. The application should make clear that these are enabling awards to expand inclusion to members of underrepresented groups that lack the resources to participate without the funds.
   b. **Offer grants to early career scholars with financial need,** e.g., students, postdoctoral scholars, and pre-tenure faculty. The application should make clear that these are enabling awards to expand inclusion to those early career scholars that lack the resources to participate. Since some institutions have pots of funding dedicated to early career conference participation, consider asking applicants to list any additional sources of funding they have (e.g., department travel award). For faculty (e.g., if you include pre-tenure faculty), you can request they list their ‘current

& pending support'. Include optional space for them to address their support levels, e.g., if the faculty member has a large lab, travel funds may be spread thin and thus, funding is needed. Or, the faculty member may wish to attend your conference that is out of their 'core research area', and thus, seek cross-field funding. These data can be used in the selection process to determine where limited funds are allocated.

c. **Include a "financial need" funding category** with unrestricted funds. Some participants will not qualify under standard award categories due to, for example, career stage or citizenship status. Consider offering funding possibilities to those that express financial need, e.g., a partial or full registration waiver. Societies will need to determine eligibility. See 'inclusive fundraising'.
d. **Avoid offering awards only as reimbursements**, especially for those participants that are early-career, minoritized, and/or from a developing country, PUI, or HBCU/1890. Offer options that ensure recipients aren't out of pocket for expenses, especially if they must wait weeks or months for reimbursement (i.e., directly defray fees or lodging). Consider paying directly for travel costs (i.e., booking flights). Give awardees the option to choose from possible ways to receive the awards (e.g., waived registration, travel costs, lodging).

3. Inclusive Fundraising
    a. **Solicit unrestricted funds**. Conference awards are often derived from restricted sources such as government grants that can be used only for specific purposes, e.g., travel costs for specific speakers, or early career participants with specific citizenship. Unrestricted funds enable broader inclusion; these are funds that aren't earmarked for specific purposes or people. Societies can use unrestricted funds to offer "financial need" awards for those that don't otherwise qualify for funding. Funding could be further organized into, e.g., "early career", "global South", "parents of young children", or "general financial need" support. Unrestricted funds are commonly provided by commercial or non-governmental sources such as journals, industry groups, or societies that are relevant to your community and you have relationships with.
    b. **Develop a short fundraising 'pitch' that includes the conference's draft program, your 'ask' (e.g., sponsorship), and what, if anything, the sponsor will receive** (e.g., named as a session sponsor; inclusion of their advertising flier in materials). It's often

effective for fundraising requests to come from a personal connection (e.g., an organizer who is a journal editor or reviewer could contact journal staff or leadership directly.) Make requests at least 6-12 months before the conference; many groups commit their budgets on an annual basis.

c. **Establish an "Accessibility Fund" to enable inclusive participation**. If your conference supplies transportation (e.g., shuttles from lodging to conference site), the best practice is to ensure broad accessibility, such as wheelchair accessibility. If this is not possible, make clear on the conference accessibility webpage that provided transportation lacks wheelchair accessibility and participants with additional needs can apply for accessibility funds (e.g. Uber, taxi). Make the application simple.

d. **Consider adding a small optional fee during registration for attendees to support inclusive participation**. Such an 'equity/accessibility fund' could be used to support participation for those with financial need or don't qualify for other sources. Ensure that the optional added fee is clearly marked as a separate 'donation to equity/accessibility fund' on the receipt, as it may not be reimbursable by certain funding sources. Consider exploring alternate ways to receive donations so that they're clearly separated from conference costs (e.g., Venmo, PayPal, and the like).

4. Conference lodging and travel
   a. **Establish sufficient lower cost budget housing options**. Consider contracting with local universities that offer inexpensive summer student housing, if relevant to your meeting dates. Make sure the housing is accessible and provides adequate amenities (e.g., proper bed for those with physical disabilities, microwave/refrigerator for those on a budget, with a need to store medicine, and/or with specific eating habits, etc.)
   b. **Seek a range of low- to mid-priced hotel options that have decent ratings**. Consider arranging room blocks and discounts by contacting hotels for meeting discount codes. Ensure the block contains different types of accessible rooms (e.g., roll-in shower, hearing impaired.)
   c. **Describe clearly on your website the various pricing levels of lodging**, e.g., budget, moderate, higher end. Include important information such as: distance from the venue, proximity to public transportation; estimated time to walk to the venue; taxi, Uber, Lyft, etc. availability and estimated costs for traveling to the conference venue.

d. **Provide “roommate matching” for attendees that book lodging via the conference portal**. This can occur during the online registration process where attendees may opt-in to share a room with two beds, typically with someone of the same gender. This approach will likely require management. A less hands-on approach is to set up a communication system to allow attendees to seek roommates and manage their own arrangements. This approach comes with some risk as the individuals are unknown to each other, and relationships and finances are managed by them.

e. **Accessible transportation:** Ensure that conference lodging is accessible by public transportation and/or within walking distance of the main venue. Provide “transportation matching” for to/from the airport, particularly if public transportation is less available or reliable. This could be arranged in advance of the meeting to coordinate arrival at the nearest local airport (as applicable) where participants meet at a designated spot at the airport arrival, and take a taxi/Uber/Lyft to the conference
site or the common lodging site. This could be arranged informally or formally at the conference for the final day (e.g., set up an easel with times for people to sign up and travel together.) Set the expectations and guidelines clearly as to how payments are to be handled, particularly if it is to be done by the participants themselves (without organizer mediation.)

f. **Volunteers to help at the conference in exchange for funding support.** Consider if there are appropriate conference tasks that could be taken on by some attendees in exchange for free or reduced conference fees. For example, local students, postdocs, or staff may know the area well enough to serve as on-site guides before, or between, sessions. Or, helping pass around microphones during the Q&A sessions; distributing materials at the registration desk; or assembling conference materials before the meeting starts. Ideally, helpers aren’t required to work during sessions so that they can participate fully, opportunities are spread across labs, and expectations and compensation should be transparent at the point of invitation. Additionally, the work hours should be reasonable for the amount of funding provided. A best practice is to develop a ‘contract’ outlining the expectations of the volunteer and the organizing committee (i.e., what the volunteer is committing to do, and what they receive in exchange, and the consequences if either party doesn’t fulfill their obligations). This contract should be agreed upon in advance, and signed by both the organizer and the volunteer, and

each retains copies. Each society should determine if they want to establish an enforcement mechanism /process.

5. Food at conferences
    a. **Provide affordable options for meals not included in the conference fee.** Most conferences include at least some food within the registration fee. However, many do not fully cover meals throughout the conference, and meetings in high-cost areas present additional challenges to many participants. Provide a map, list, and recommendations for a variety of lower cost restaurants and grocery stores within walking distance of the venue, especially if meals during the conference are not included and participants are expected to fund themselves.
    b. Make clear on the conference website well in advance the meals to be included in the registration fee and those that are the responsibility of participants. Link this information to the list of lower-priced stores and restaurants that you developed.
6. Hybrid option
    a. Conference participation is expensive, especially for attendees that must travel to the site and stay in lodging. Beyond on-site costs are fees such as to secure travel documents, take time off from work, possibly unpaid, or arrange for caregivers for family members. Hybrid meetings have recently been widely adopted in response to the global Covid-19 pandemic and have expanded access to many people that otherwise would be unable to attend.
    b. Similar to accessibility, hybrid components should be planned carefully and early and not as an afterthought. Resources (time and money) are required to provide a worthwhile virtual experience; societies likely will need to consider possible features and prioritize them. Engaging your community in transparent discussions of costs and options can help guide your choices and increase goodwill. Ideally, open discussion will increase community buy-in and perhaps creative problem-solving. Ensure that accessibility features are part of the discussion.

(2) PHYSICAL ENVIRONMENT

The physical environment of the conference includes the conference venue (where presentations and the scientific and social events will take place) as well as the conference hotel or other overnight lodgings; attendees will need to travel between and navigate these spaces during the conference, so it is imperative that organizers assess the continuum of these physical spaces to

ensure accessibility.

1. Communication of accessibility arrangements
    a. Accessibility arrangements should be clearly detailed on the conference website so attendees can plan for their individual needs and assess their risks/safety.
    b. Provide an accessible conference map, including directions for drivers, cyclists, walking, and public transit, wayfinding for wheelchair accessibility including elevators, accessible bathrooms and entrances, and locations of specialized rooms, such as lactation room and quiet room. Ensure that the map itself is accessible (by a screen reader, etc.); if provided as a PDF, add alt-text.
    c. Images of main meeting rooms. These can be emailed or otherwise made available to attendees before the conference starts and/or included in the conference map. Also useful is to include this information on a conference app (for phone or tablet use), which is becoming more common.
    d. Offer a buddy system
        i. This could be a sign-up sheet to help participants match up with other attendees with disabilities, OR pay students to pair with participants who require physical help/guidance around the conference for specific events like mixers
        ii. Other ways that the buddy system could increase accessibility is through improving safety, such as escorts to/from restaurants/bars at night or pairing people for early morning workouts/running
        iii. Consider developing a way to vet potential buddies, and make it clear that all interactions between buddies still fall under the Community Agreement
    e. Provide the necessary accommodations for service animals, such as a place for the animals to relieve themselves, and contact information for a local vet.
2. Site visit
    a. The local committee should walk the venue and ensure that it is accessible to those in wheelchairs and/or those with low-mobility. To ensure that there is adequate assessment, request that the venue hosts prepare, in advance of a site visit, documentation of the venue's accessibility features. Use that list as a starting point to discuss internally and with venue staff whether accessibility is addressed.
    b. Prior to signing contracts, discuss with the site management the available accessibility features, including what is required by local laws (e.g., ADA compliance in the USA)

c. Appoint a local accessibility chairperson for the conference
d. Provide secure storage (for items people with disabilities may have to carry)
e. Ensure that poster session aisles are wide enough for those with low-mobility or who use wheelchairs or other mobility devices; consider making aisles "one-way" for easier navigation
f. Accessible auto and bicycle parking near the venue
g. Ensure that if there is a stage for speakers, that there is a ramp for those with mobility issues
h. Ensure adequate signage for attendees that use wheelchairs so that they can easily navigate safely from conference lodging and local transportation (if relevant) to the meeting site. Include signage within the conference venue so that attendees can easily find elevators or other assistance.
i. Examine the lactation room and ensure that it meets the basic requirements (outlined in Family Environment below)

3. Two quiet/low stimulation rooms (for those with "invisible wounds", such as survivors of trauma, individuals with neurological conditions, and anyone who needs a break). One room is for quiet & calm, the second (if needed) for participants that may need to have accommodation for potentially disruptive behaviors (physical or verbal outbursts)
4. Accessible seating clearly labeled along aisles in every room with seating
    a. Furniture with and without arms
    b. Chairs that aren't connected to other chairs so seat placement can be adjusted when needed.
    c. Furniture for people of various sizes
    d. Be able to reserve aisle seating for people who may need an easy exit
    e. Aisles left clear and at least 36" wide for wheelchair accessibility
    f. Priority seating (labeled) at front of rooms for individuals who are hard-of-hearing, have low-vision, are deaf and need to be near interpreters, and so forth
    g. Some rows with open spaces without chairs where a wheelchair could go, rather than having to move chairs or put the wheelchair in an aisle

(3) AUDIO/VISUAL ENVIRONMENT

Worldwide, 8.5% of people have some form of hearing loss and/or vision impairment. In the United States, 30% of working professionals have an audio/visual disability (20% are deaf or hard of hearing and 10% have some degree of vision loss) (https://www.3playmedia.com/accessibility-online-video-

stats/). This is a considerable number of people who require some type of accommodation in order to benefit fully from conference participation. Organizers should survey their community well in advance to determine accessibility needs that should be addressed. For conferences to be accessible to this population, we provide various ways to accommodate different degrees and types of audio/visual needs for both in-person and remote attendance.

1. The conference website should follow the W3C Recommendation from the Web Content Accessibility Guidelines (WCAG) 2.0 and be tested for functionality before sharing (https://www.w3.org/TR/WCAG20/).
2. Several microphones should be available in every conference room. Additionally, we recommend providing headsets that link directly into the A/V system. Survey participants during registration so that enough headsets are made available.
3. Consider providing sign language services on-site (and include good lighting so the audience can see the person signing) or remotely. We recommend asking participants about their needs during the registration process so organizers know in advance the accommodations that are needed, and can address them by sourcing local interpreting agencies ahead of time in order to better anticipate costs.
4. Live caption writer on site and/or remotely.
5. CART captioning (in the room and online) (https://www.hearingloss.org/hearing-help/technology/cartcaptioning/).
6. For international conferences, we recommend providing access to the conference webpage and documentation on a variety of platforms, taking into account some countries' webpage restrictions. For example, in China, where many common online platforms are restricted, WeChat is broadly used for communication. An additional consideration would be translating essential information on local travel and accessing the conference in different languages. One possible resource is local tourist information as cities usually have information available in different languages. If it is not feasible to translate the webpage or documentation, we recommend informing conference attendees that the automatic Google translator can be used on most web pages; alternatively, they can copy and paste information into DeepL, a free online translator: https://www.deepl.com/translator.
7. Offer an online workshop several weeks to a month before the conference to provide guidelines on how to make their presentation accessible (see Appendix A). This will benefit all conference attendees. Make this information available in written and downloadable

format on the conference site at the earliest stages of meeting development.

8. The conference program should be available in various formats (print, electronic, in large print, and if the need is indicated, Braille).
9. Provide an assistant to help advance slides for presenters who may have visual impairments.
10. Reserve ground floor rooms or rooms near elevators. Ideally, rooms will have Braille labeling.
11. Provide an assistant who can read posters for blind/low vision attendees and/or provide tactile maps/audio options for posters.
12. Consider a way to submit questions to speakers electronically for in-person meetings. Slido is an application that can accommodate this (https://www.slido.com/).

(4) FAMILY ENVIRONMENT

Caregivers are a major part of the workforce, and conferences get higher attendance rates when they offer accommodations to bring children along (https://doi.org/10.1073/pnas.1803153115). The so-called “baby penalty” (www.chronicle.com/article/The-Baby-Penalty/140813) can affect career development at any stage. Here we include recommendations that could help make conferences more inclusive for caregivers with children of all ages. We point to conference characteristics that would help ensure that the conference is family-friendly and inclusive to caregivers, and provide resources they need to take care of their children. Additionally, we recommend that offering a virtual meeting component could make it more accessible to caregivers who cannot or decide not to travel.

2. Ensure that a **lactation room** is available at the conference venue. The lactation room should be clearly marked and included in the conference information, and should not be simply a closet or space in a bathroom. Consider the following recommendations (See Appendix B).
    a. If a lactation room is unavailable at the venue, we recommend bringing a portable lactation space to the venue, e.g., https://www.mamava.com/all-products or https://www.nestl-space.com/. Make sure that the portable lactation space is located outside of busy areas, to provide the user with privacy and a comfortable environment.
    b. It is important that all conference organizers and staff know what a lactation room is and where it is located. Make sure the room is clearly identified and procedures for its usage (e.g., reservations) are

established and communicated to attendees and staff.

   c. Some parents may not be able to schedule the use of the lactation room around all talks and events. If possible, we recommend having streaming of the talks available in the lactation room.

3. Offering options to help caregivers with **childcare** during the conference is essential. There are several options, from offering childcare on-site, to providing grants for caregivers to bring a caregiver.
   a. Some options for professional companies that offer childcare on-site: https://conferencechildcare.com or https://www.kiddiecorp.com/ or https://ahelpinghandevents.com/.
   b. For caregivers that attend the conference with children, attending every session might be impossible. Consider offering partial registration for parents with children on site might enable broader attendance. It may include registration for individual days, or morning/afternoon shifts.
   c. Even for local scientists, childcare tends to be unavailable or complicated to find during weekends. Consider scheduling conferences from Monday to Friday, during regular working hours.
   d. Childcare is harder to find and schedules are hard to maintain, especially in a foreign city or country. To take into account the dependents' jet lag and that changes in routines might make the life of caregivers harder, we suggest not to schedule major events (like plenary sessions) in the early morning or evening.
   e. Similarly, allow presenters with attending children to schedule their talks at times that would better accommodate their family obligations. You can survey invited speakers in advance to help structure the program; if the program is already established you could survey speakers to determine if there are caregivers (or speakers from disparate time zones) to help decide speaker order to best accommodate them.
4. Ensuring that the conference is **welcoming for caregivers and children** will provide a better and more inclusive experience. Here we provide recommendations to help create an inclusive environment:
   a. State in conference materials (web page and abstract book) that the conference welcomes children (in non-disruptive ways).
   b. Provide official registration badges for kids with a fun job title (like "Future scientist").
   c. Provide a room designated as a "parent lounge" where families can spend some time with their children, and interact and connect

with other families.

d. State on the conference web page and abstract book that babycaring is welcomed during the conference.
e. Connecting with other parents in a similar situation can strengthen belonging. We encourage conference organizers to create a social group in advance for caregivers with children that are attending the conference.
f. Networking is an essential part of any conference. To fully include caregivers in these events, we recommend including social events for families and/or events that are family friendly with respect to location and timing.

(5) WELLNESS ENVIRONMENT

Wellness can be an all-encompassing term used to describe one's physical, mental, and emotional wellbeing. In the context of developing inclusive conferences, preparations can be made to promote the safety and comfort of all participants, including those who are often excluded from conferences because they require particular arrangements and may not otherwise have the physical and/or financial ability to attend the conference. Establishing protocols for catering, social events and social conduct/interaction, COVID and other health concerns, and preferences for interaction, will encourage participation from a wider community and will benefit the community overall. When these recommendations cannot be met, clearly indicate the information on the website so participants can plan accordingly. Some participants may need to make individual arrangements, some of which should be financially supported by the conference through a "conference accessibility fund".

1. Catering
    a. Make arrangements to accommodate various dietary restrictions, and consider cross-contamination as participants may not be able to eat from buffet settings. Survey your participants during registration about their specific needs.
        i. Please see Appendix: Wellness for a list of examples
    b. Provide a complete ingredient list so attendees can assess if foods follow individual dietary restrictions.
    c. Participant mobility can be increased by clearly blocking off the food area(s) away from crowds so low-mobility participants and participants with wheelchairs do not have to go through crowds. In addition, place the food area near the entrance so that participants do not have to navigate between tables and chairs to access.
    d. Consider providing additional catered events for low-income

students who rely on catered events to obtain meals during the conference.

2. Social events
    a. Host events where affinity groups can create a sense of belonging and build community such as ethnic group socials, gender identity and sexual orientation socials, family socials for those attending with children, or other groups represented in the conference population. Allow individuals or groups to host their own event and provide financial support such as for catering to encourage participation.
    b. Host at least one non-alcoholic social such as a dessert bar or ice cream social, arts and crafts social.
    c. Consider holding a “movement” event. This can be a single event such as the “Weed Stampede 5K Fun Run/Walk” at North American Arabidopsis conferences. You might also provide yoga mats in a separate quiet room and encourage participants to use them for stretching. Consider arranging for a volunteer (e.g., from the organizing committee or a participant) to lead a yoga session open to all and at no cost to participants. This could be set up in the morning prior to sessions and/or during the day if there are breaks.
3. COVID and health beyond
    a. The conference website and the on-site registration desk should include information on where to locate first aid on-site, the nearest hospital, and local drug stores.
    b. Provide plenty of good quality masks (e.g., N95s).
    c. Conference rooms should provide excellent ventilation
    d. Conference rooms should provide CO_2 monitors, and capacity limits for optimal indoor air quality are posted
    e. Provide multiple options for outdoor space (especially for eating)
    f. Provide hand sanitizer, especially around events with buffet style
4. Preferred method of communication/interaction – Provide name tags/banners for users to indicate:
    a. Shyness/socialization comfort
    b. Physical contact comfort (shake hands/no touching/hug etc.)
    c. Pronouns
    d. Stimulation level (e.g., “easily overstimulated”)
    e. Text on name tags should be large enough to be seen from 3 feet away and printed in high-contrast (e.g., black text on white paper)
    f. Consider providing different options for how to wear name tags (hanging from neck, pinned to shirt)
5. Hybrid option - offering a virtual component will enable access to the

meeting for many groups such as people with families, disabilities, financial need, visa and travel concerns

a. Provide live captioning by hiring a real-time live captioner for main events such as keynote speakers
b. Assign a moderator for talks to monitor and repeat questions, and provide clarifications
c. Encourage closed-captioning for all recorded and live talks
d. Host virtual social events for off-site participants

Appendix A: Presentation Guidelines

Both in-person and online presentations

- Title on each slide to summarize main point
- Presentations, including figures, should use colors that are colorblind-friendly (https://davidmathlogic.com/colorblind/)
- Spell out acronyms (don't just verbalize them)
- Provide pdfs of the presentation beforehand (if possible)
- Video captioning that can either be transcribed by a person or edited later (automatic video captioning typically has many errors)
- Presenters should describe the slides, pictures, and videos for those with visual impairments
- Use large font (40pt for in-person meetings and at least 24pt for virtual meetings)
- Use a sans serif font (like Arial, Verdana, Helvetica)
- Provide alt text and/or image descriptions
- Limit amount of text on slides
- Speak clearly and always use a microphone
- Face the audience when speaking for those who rely on lip reading
- Use plain language
- Avoid using idioms and other phrases that are not well known to speakers of other languages and/or people with some cognitive or developmental disabilities
- Limit the number of visuals on a slide
- All graphs/charts should be described and summarized
- Give the audience time to read your slides
- Use the built in accessibility checks that Microsoft powerpoint and other presentation builders typically have
- Presentations/workshops that last longer than 45 minutes should include a break
    - If you are including breaks let the participants know ahead of time how many and approximately when they will occur. Likewise if you are not providing built in breaks, let the participants know so that they can decide to take their own as needed.

Online only

- Turn on your video and center your face so the audience can see your lips while you talk and read your facial expressions
- Minimize background noise
- Record the talk
- Blur the background of your video
- Add name and pronouns in your profile
- Use a good quality microphone

Appendix B: Lactation room characteristics

- Ensure that the designated lactation room is accessible with a wheelchair and large enough to accommodate all needs.
- Ensure that the lactation room is located in a quiet space, and avoid passage areas.
- Do not locate it in a restroom or space that can only be accessed through bathrooms, locker rooms, or similar areas; lactation rooms in restrooms do not meet the requirements of the Affordable Care Act (ACA)
- Do your best to provide a sink, so breast pump equipment can be cleaned, and install a regular faucet instead of a motion-sensing faucet, which shuts off too frequently for effective cleaning
- Offer a microwave, so breast pump equipment can be sterilized
- Make clear access to an electric outlet
- Obtain a washable, comfortable chair
- Place a small table next to the chair
- Consider using a non-carpeted floor that can be easily cleaned
- Offer paper towels and sanitizing wipes
- Arrange for the room to be placed on the building's regular cleaning schedule
- Identify the room with a lactation space sign
- Create a locked or secure area, and provide a privacy please sign
- Assure that the room is temperature-controlled, warm and comfortable
- Post a room usage schedule, or create a Google or Outlook calendar so those who use the room can view and edit
- Consider providing lockers for storing personal items like pumps, bottles, and parts.

Appendix C: Economic Accessibility

- Site selection: while developing options consider location costs: travel, lodging, food for participants from a variety of career stages, institution types, and geographic locations attending.
- Conference budget: interrogate registration fee development and consider who (speakers, organizers) are covered. Avoid pricey activities in registration fees and/or raise external funds to support them. Ticketed activities: develop tiered costs. If catering is expensive, consider reducing food provided (to lower the fee) and map out nearby, accessible, diverse, low-cost food options.
    - Registration fees: Tiered fees to reflect varying resource levels, e.g., by career stage whereby faculty contribute higher fees and subsidize students and postdocs. Also consider: participants from less-resourced countries and institutions (e.g., PUIs, Global South).
    - Offer free or discount registration fees to participants with financial need in exchange for reasonable volunteer work.
    - Develop an external (to registration revenue) fundraising plan to support those with the most need (e.g., early career, minoritized, parents with young children, those with disabilities, etc.)
    - Solicit restricted funds (e.g., government grants) to support some participants and unrestricted funds from companies, societies, and journals, for more inclusive funding.
    - Develop non-onerous funding applications that accommodate a variety of less- resourced participants. Offer funding options that do not rely on reimbursement, especially for those with fewer resources.
- Lodging: Secure a range of decent lodging options near the venue, or easily accessible by public transportation. Clearly communicate the price range, amenities, and proximity to the venue.
    - Inquire if there are local colleges or universities that may offer low-cost residence halls.
    - Facilitate 'roommate matching' to allow participants to share lodging costs.
- Communicate clearly your accessibility efforts:
    - Identify on conference materials the locations of easily accessible & affordable restaurants & grocery stores to obtain budget meals (for meals not supplied by the meeting).
    - Communicate clearly on the website what is included in the registration fee, e.g., meals, coffee, and what must be budgeted separately by participants.
- Develop a hybrid option that enables meaningful participation; price the hybrid fees as low as possible (you may need external fund-raising to keep fees reasonable)

Appendix D: Wellness Accessibility - Catering

- Vegan
- Vegetarian
- Diabetic (make non-sugar beverages and foods available)
- Low salt
- Allergies
  - Celiac-safe
  - Gluten-free
  - Nut-free
  - Peanut-free
  - Soy-free
  - Fish-free
  - Shellfish-free
  - Egg-free
  - Lactose-free
  - Wheat-free
- Religious dietary requirements
  - Hallal
  - Kosher
- Low sensory
- Ensure that non-caffeinated drinks are available and clearly marked
- Have water bottle filling stations available throughout venue
